# Erythroblastic Island Macrophages Shape Normal Erythropoiesis and Drive Associated Disorders in Erythroid Hematopoietic Diseases

**DOI:** 10.3389/fcell.2020.613885

**Published:** 2021-02-12

**Authors:** Wei Li, Rongqun Guo, Yongping Song, Zhongxing Jiang

**Affiliations:** Department of Hematology, The First Affiliated Hospital of Zhengzhou University, Zhengzhou, China

**Keywords:** EBI macrophages, erythropoiesis, central macrophages, EPOR, erythroid hematopoietic disorders, β-thalassemia, polycythemia vera

## Abstract

Erythroblastic islands (EBIs), discovered more than 60 years ago, are specialized microenvironments for erythropoiesis. This island consists of a central macrophage with surrounding developing erythroid cells. EBI macrophages have received intense interest in the verifications of the supporting erythropoiesis hypothesis. Most of these investigations have focused on the identification and functional analyses of EBI macrophages, yielding significant progresses in identifying and isolating EBI macrophages, as well as verifying the potential roles of EBI macrophages in erythropoiesis. EBI macrophages express erythropoietin receptor (Epor) both in mouse and human, and Epo acts on both erythroid cells and EBI macrophages simultaneously in the niche, thereby promoting erythropoiesis. Impaired Epor signaling in splenic niche macrophages significantly inhibit the differentiation of stress erythroid progenitors. Moreover, accumulating evidence suggests that EBI macrophage dysfunction may lead to certain erythroid hematological disorders. In this review, the heterogeneity, identification, and functions of EBI macrophages during erythropoiesis under both steady-state and stress conditions are outlined. By reviewing the historical data, we discuss the influence of EBI macrophages on erythroid hematopoietic disorders and propose a new hypothesis that erythroid hematopoietic disorders are driven by EBI macrophages.

## Introduction

“Erythropoiesis is a process by which hematopoietic stem cells (HSCs) proliferate and differentiate via multiple distinct developmental stages, to eventually generate mature red blood cells (RBCs)” (Yan et al., 2017; Huang et al., 2018; Qu et al., 2018). This process occurs at the erythroblastic island (EBI), which is composed of a central macrophage surrounded by developing erythroid cells (BESSIS, 1958). EBI, first described by Bessis in 1958 (BESSIS, 1958), was functionally validated by Mohandas and Prenant (1978), who showed that the number of EBIs was significantly decreased in hypertransfused rat bone marrow (BM) (Mohandas and Prenant, 1978). The roles of macrophages in supporting erythropoiesis have also been demonstrated *in vitro*. For example, macrophages were found to promote proliferation/survival of erythroblasts (Hanspal and Hanspal, 1994; Rhodes et al., 2008). On the other hand, abnormal macrophage differentiation in *EMP*-null (Soni et al., 2006) and *KLF1*-null mice (Porcu et al., 2011) reportedly led to significantly impaired erythropoiesis and anemia. Depletion of macrophages with either clodronate liposomes (Ramos et al., 2013) or with CD169-diptheria toxin (Chow et al., 2013) provides direct evidence that macrophages play significant roles in erythropoiesis *in vivo*, particularly under stress conditions. Nevertheless, the contribution of EBI macrophages to normal, as well as disordered erythropoiesis, remains unclear, owing to the non-selective nature of approaches used in deleting macrophages in previous studies. This is largely attributed to the inability to isolate EBI macrophages for cellular and molecular studies, indicating a need for accurate identification of EBI macrophages and guide development of novel approaches for their isolation.

Numerous research efforts have been made in identifying the possible proteins expressed by EBI macrophages, such as erythroblast macrophage protein (EMP), vascular cell adhesion molecule-1 (VCAM1), erythroid Krüppel-like factor 1 (KLF1), and DNase2α, among others (Soni et al., 2006; Porcu et al., 2011; Xue et al., 2014). However, the mechanism through which EBI macrophages regulate normal erythropoiesis and erythroid hematopoietic disorders remains unknown, owing to a lack of accurate phenotypic profiles of these macrophages. Recently, An's group used the *Epor*-eGFPcre mouse model and a specific antibody against human EPOR, to characterize EBI macrophages (Li et al., 2019). In addition, they performed RNA sequence of the newly identified Epor^+^ EBI macrophages to reveal specialized functions and potential molecular mechanisms of EBI macrophages in supporting erythropoiesis. These results indicated that EBI macrophages promote erythropoiesis via directly interacting with erythroblasts, secreting growth factors, phagocytosing senescent RBCs, providing iron, and finally engulfing nuclei enucleated by erythroblasts (Li et al., 2019). Furthermore, findings from Paulson's group demonstrated that Epo/stat5 signaling may promote stress erythroid progenitor (SEP) transition, from proliferation to differentiation, by acting on Epor^+^ macrophages in a splenic niche (Chen et al., 2020). In this review, we comprehensively described the role of EBI macrophages in regulating normal erythropoiesis and erythroid hematopoietic disorders based on historical data related to their discovery, heterogeneity, identification, and functional characteristics. We further highlight significant unanswered questions in view of providing vital knowledge to guide understanding of this tightly regulated process in normal and disordered erythropoiesis of hematologic diseases such as β-thalassemia and polycythemia vera (PV), among others.

## Distinct EBI Types Reveal Heterogeneity of EBI Macrophages

**Figure 1:**
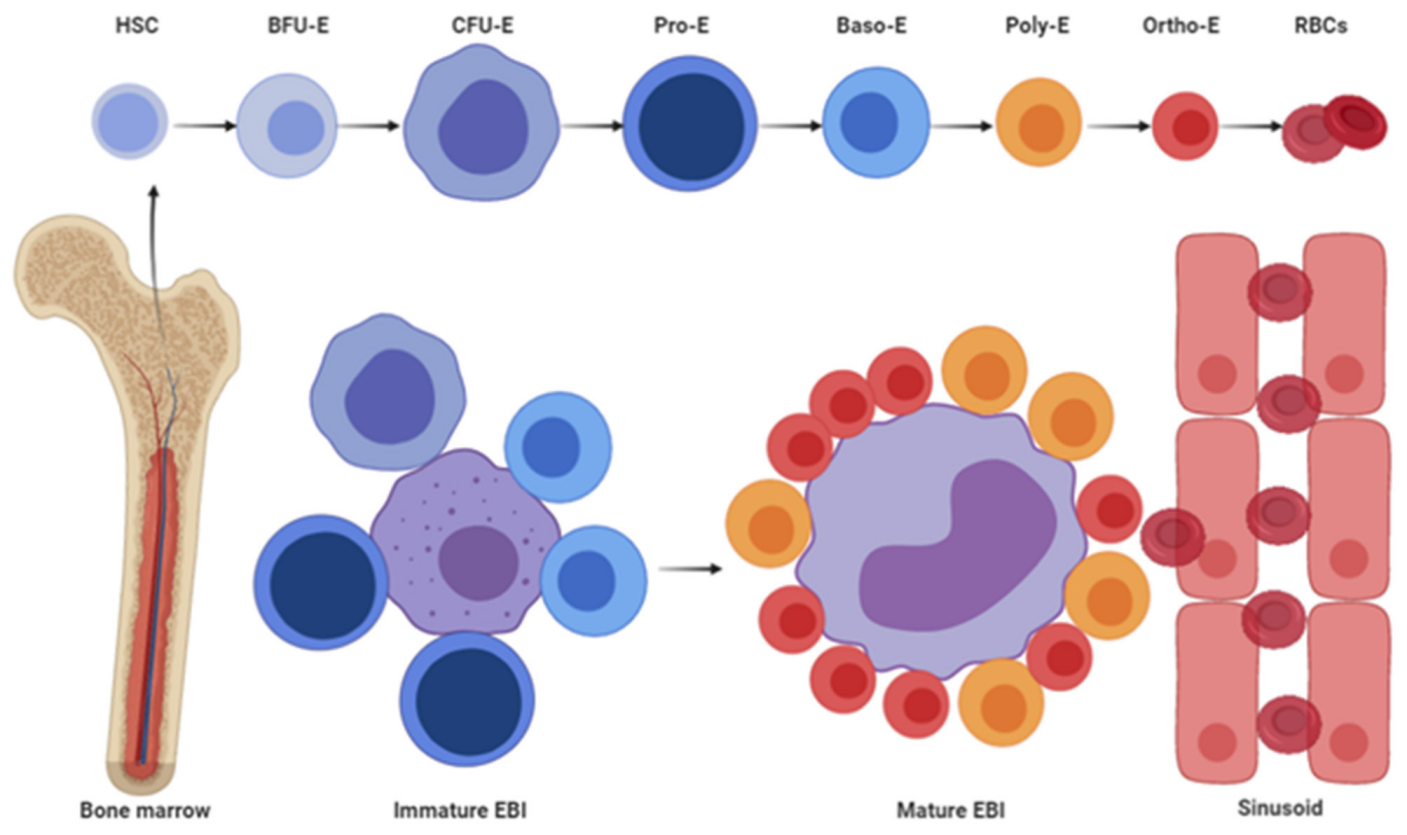
Schematic diagram of two types of EBIs and the process of EBI maturation.

The EBI, composed of a central macrophage and surrounding erythroid cells, was the first hematopoietic niche discovered by Marcel Bessis in (1958). Since the discovery, many studies have been focused on the morphological characteristics. For example, electron microscopy-based studies, between 2003 and 2016, have revealed two distinct types of EBI, which differ by size, morphology, attachment based on different developmental stages of erythroid cells, and location relative to sinusoid (Yokoyama et al., 2003; Yeo et al., 2016). The first type, termed immature EBI, comprises a small-sized, domed macrophage attached with early-stage erythroid cells and away from sinusoid, whereas the other, known as mature EBI, is composed of a large and flat macrophage that is attached by late-stage erythroblasts and close to sinusoid. Interestingly, immature EBI mainly comprises early-stage erythroblasts, whereas mature EBI has erythroblasts at late stage. Early-stage erythroid progenitors and erythroblasts are responsive to interleukin-3 (IL-3), stem cell factor (SCF), and erythropoietin (EPO) to maintain their self-renewal, proliferation, and differentiation, whereas late-stage erythroblasts can no longer proliferate. In fact, the latter need to enucleate the condensed nuclei and import iron to enable hemoglobin synthesis (Dzierzak and Philipsen, 2013). EBI macrophages play distinct roles in RBC development across different developmental stages, from early-stage erythroblasts to late-stage erythroblasts. Specifically, immature EBI macrophages secrete growth factors that promote proliferation and differentiation of early-stage erythroblasts (Hanspal and Hanspal, 1994; Rhodes et al., 2008), whereas mature EBI macrophages promote maturation of late stage erythroblasts by providing iron and phagocytosing nuclei (Donovan et al., 2005; Miyanishi et al., 2007; Chow et al., 2013; Ramos et al., 2013). During maturation, EBIs migrate toward sinusoid (Yokoyama et al., 2003). Previous studies have shown that sinusoid endothelial cells serve as niches for HSCs and megakaryocyte progenitors (Avecilla et al., 2004; Pitchford et al., 2012; Crane et al., 2017); however, the roles of these endothelial cells in supporting erythropoiesis have been largely unknown. Additionally, endothelial cells from different sources have been shown to promote macrophage differentiation and polarization to M2-like macrophages, whereas the endothelium in fetal liver (FL) regulates the seeding of tissue-resident macrophages via a membrane protein (He et al., 2012; Rantakari et al., 2016). EBI macrophages are M2-like macrophages population (Heideveld et al., 2018). Thus, it is possible that SECs may play important roles in erythropoiesis by influencing migration and/or maturation of EBI macrophages and EBIs. A schematic representation of the two EBI types (Figure 1) indicates that they play distinct roles in RBC production. During erythroid cells maturation, EBIs migrate to the sinusoid, and mature erythrocytes are released into peripheral blood. Taken together, there are two distinct EBIs identified by researchers, which suggest EBI macrophages are heterogeneous.

EBI macrophages, which act as nurse cells for erythroblasts and form a structure called EBI, were the first ever identified BM resident macrophages (BESSIS, 1958). The interaction between central macrophage and erythroblasts within the EBI is crucial for erythroblast survival and maturation, which then generates functional enucleated reticulocytes to maintain homeostasis. Importantly, these macrophages promote erythroid maturation by producing growth factors, providing iron for hemoglobin synthesis, and clearing extruded nuclei (Chasis and Mohandas, 2008; Vinchi, 2018). However, it is still unclear whether these functions in supporting erythropoiesis come from distinct subpopulations of EBI macrophages or not. To address this question, multispectral imaging flow cytometry (IFC, also called imagestream) and flow cytometry–based findings from Kalfa's group, Paulson's group, and An's group were concluded, showing that EBI macrophages are heterogeneous (Seu et al., 2017; Liao et al., 2018b; Li et al., 2019). Kalfa's group (Seu et al., 2017) used imagestream to show heterogeneous expression of Vcam1, F4/80, and CD169 by EBI macrophages within the EBIs. In their study, they found that the distribution of CD169 is heterogeneous on BM EBI macrophages but not expressed by FL EBI macrophages (Seu et al., 2017). In addition, CD11b was previously used to identify EBI macrophages by flow cytometry (Jacobsen et al., 2014), but not detected on the BM F4/80^+^ EBI macrophages (Seu et al., 2017). Paulson's group reported that CD11b^+^Ly6C^+^ “monocyte-like” macrophages expressed low-level F4/80, CD169, and Vcam1, which interacted with immature CD133^+^Kit^+^ SEPs. However, CD11b^+^Ly6C^−^Pre-RPMs and CD11b^low^Ly6C^−^RPMs are interacted with Ter119^+^ erythroblasts, which expressed CD169 and Vcam1 in BM transplantation (BMT) model (Liao et al., 2018b). Study from An's group indicated that more than 90% of EBIs are formed by F4/80^+^Epor^+^macrophages in mouse BM, FL, and stress SP niche (Li et al., 2019). Flow cytometry and imagestream results revealed that Vcam1 and CD169 are broadly distributed on EBI macrophages, although only a proportion of EBI macrophages express CD163 and Ly6C (Li et al., 2019) in mouse BM. CD11b is moderately distributed on mouse BM EBI macrophages from both flow cytometry and imagestream results, suggesting that EBIs are formed by both CD11b^+^ and CD11b^−^ macrophages (Li et al., 2019). Significantly, Ly6G, previously implicated as an EBI macrophage marker (Jacobsen et al., 2014), is not expressed on the EBI macrophages in mouse BM, FL, and SP (Li et al., 2019). Overall, these findings confirmed the heterogeneity of EBI macrophages between distinct hematopoietic organs and even within the same organ. As discussed above, EBI macrophages may support different stages of erythroblasts via distinct functions. Thus, it is likely that the supporting roles may be due to the distinct subpopulations of EBI macrophages. Future studies are expected to use single-cell RNA-seq analyses to further characterize these subpopulations. This enabled us to study distinct roles of EBI macrophages in supporting erythropoiesis and thereby to answer questions around the heterogeneity of EBI macrophages.

## Identification of EBI Macrophages and Characterization of Their Potential ROLES During Erythropoiesis

### Identification of EBI Macrophages and Functions of Adhesion Molecules Involved in EBI Formation

Pioneering works by many groups characterized the possible molecules expressed by EBI macrophages, which include EMP, Vcam1, CD169, αV integrin, Klf1, Dnase2α, and so on (Sadahira et al., 1995; Kawane et al., 2001; Mankelow et al., 2004; Soni et al., 2006; Porcu et al., 2011; Chow et al., 2013). However, heterogeneity of macrophages implied that they greatly vary depending on disease state, tissue localization, and developmental origin (Bian et al., 2020). Therefore, it is likely that not all the macrophages are EBI macrophages. Based on this inherent heterogeneity, studies have attempted to identify EBI macrophages. For instance, Kalfa's group (Seu et al., 2017) employed imagestream, which combines high-throughput advantages of flow cytometry with morphological and fluorescence features derived from microscopy, to analyze EBI macrophages and associated cells within the EBI. They quantitatively analyzed EBIs, as well as their structural, morphological details and associated cells. Their study showed that EBI macrophages express several surface markers, including Vcam1, F4/80, and CD169. Based on this, F4/80, Vcam1, and CD169 were considered as the best combination of surface markers to identify mouse EBI macrophages. To examine whether all F4/80^+^Vcam1^+^CD169^+^ macrophages are EBI macrophages, An's group quantified both F4/80^+^Vcam1^+^CD169^+^macrophages and erythroblasts in mouse BM. They found that the ratio of F4/80^+^Vcam1^+^CD169^+^ macrophages vs. erythroblasts is ~1:2.6 (Li et al., 2019), given the fact that the number of erythroblasts per island ranges from 10 cells observed in rat BM (Yokoyama et al., 2002) to 5 to more than 30 erythroblasts in islands harvested from human BM (Lee et al., 1988). These findings strongly suggest that it is unlikely that all F4/80^+^Vcam1^+^CD169^+^ macrophages are EBI macrophages. Using imagestream, their study further revealed that more than 90% of EBIs are formed by Epor^+^ macrophages under both steady-state and Epo injection conditions in hematopoietic organs (Li et al., 2019). This expression was observed in EBI macrophages derived from murine BM, FL, and SP, as well as human FL, suggesting that Epor^+^ macrophages define the EBIs. Importantly, RNA-seq–based gene expression analysis of Epor^+^ and Epor^−^ macrophages from murine BM revealed that genes known to be important for the function of EBI macrophages in promoting erythropoiesis, such as adhesion molecules, *CD163, CD169*, and *Vcam1*; machinery for iron recycling, *Mertk, ferroportin, Hmox1*, and *Spic*; molecules for engulfing and digesting extruded nuclei, *Mertk* and *Dnase2*α, as well as growth factors *Igf1, Il18*, and *Vegf*β, were expected to be highly expressed in Epor^+^ macrophages, suggesting a specialized function of the Epor^+^ EBI macrophages (Li et al., 2019).

Of these many important molecules, adhesion molecules are significant for the interaction between EBI macrophages and erythroblasts. For example, only a proportion of mouse BM EBI macrophages express CD163 (Li et al., 2019), whereas most of human BM and FL EBI macrophages express CD163 (Heideveld et al., 2018; Li et al., 2019). In general, CD163 is a membrane receptor of group B scavenger receptor cysteine-rich (SRCH) family, which has previously been shown to be a receptor for hemoglobin–haptoglobin complexes and plays a role in free hemoglobin-clearing (Fabriek et al., 2007). This study also (Fabriek et al., 2007) showed that CD163 functions as an erythroblast adhesion receptor in EBI macrophages and may regulate erythropoiesis by mediating bidirectional signaling in EBIs. Akker's group reported that glucocorticoid enhances the EBI formation via increasing the surface expression of CD163 on human monocyte-derived macrophages (Heideveld et al., 2018). Intriguingly, An's group has recently documented that EBI macrophages also express Epor and that Epo enhances the ability of EBI macrophages to form EBIs both *in vivo* and *in vitro* (Li et al., 2019). Additionally, these results further showed that Epo injection also increases the expression of adhesion molecules Vcam1 and CD163, indicating that Epo may promote EBI macrophages' function in supporting erythropoiesis, at least in part by promoting the interaction between EBI macrophages and erythroid cells via increased surface expression of Vcam1 and CD163 (Li et al., 2019). Taken together, these studies suggested that CD163 might be a key adhesion molecule during EBI formation. In the future, CD163-conditional knockout (KO) on EBI macrophages should be conducted to further investigate the functional roles of CD163 expressed by EBI macrophages during erythropoiesis. In addition, glucocorticoid and EPO may work in concert to maintain efficient erythrocytes production, which are also worth to be performed.

CD169 (also known as sialoadhesin or Siglec-1) is another cell surface adhesion molecule on macrophages that belongs to the Siglec family and functions as a sialic acid receptor (Hartnell et al., 2001). Studies also showed that CD169 is extensively expressed by EBI macrophages (Li et al., 2019) and localizes at the site of macrophage/erythroblast contact in EBIs (Crocker and Gordon, 1986, 1989; Crocker et al., 1990; Morris et al., 1991). *In vivo* studies have shown that depletion of CD169^+^ macrophages in mice leads to impaired erythropoiesis, especially under stress conditions (Chow et al., 2013; Ramos et al., 2013). Other studies have reported that granulocyte colony-stimulating factor (CSF) blocks BM erythropoiesis by depleting CD169^+^ macrophages (Jacobsen et al., 2014; Tay et al., 2020). Specifically, depleting of CD169^+^ macrophages in PV mice resulted in decreased RBCs, indicating the importance of CD169^+^ macrophages during normal erythropoiesis and erythroid hematopoietic disorders (Chow et al., 2013). Although CD169 is highly expressed in EBI macrophages than non-EBI macrophages, the findings from An's group question whether CD169 expression is required for EBI formation, as EPO injection increases the quantity and quality of EBI via increased surface expression of Vcam1, as well as the percentage of CD163, but not CD169 (Li et al., 2019). In the future, more studies are needed to further unravel the role of CD169 in EBI macrophages during erythropoiesis under both steady and stress conditions.

Apart from the aforementioned works, Vcam1 (also known as CD106) is a complementary receptor for erythroid cells α4β1 integrin, which is expressed by EBI macrophages (Seu et al., 2017). In 2013, Chow et al. (2013) showed that Vcam1 is highly expressed by CD169^+^ BM macrophages, and these BM macrophages work in concert with bone morphogenetic protein 4 (BMP4)–producing CD169^+^ splenic macrophages to promote erythropoietic recovery following myeloablation. Specifically, their findings showed that antibody against Vcam1 administered after BMT impairs the recovery of BM erythroblasts, reticulocytes, and hematocrit in both macrophage-sufficient and macrophage-depleted mice (Chow et al., 2013). In 2017, Fujiwara et al. (2017) found that VCAM1 is strongly expressed in CD169^+^ macrophages in human BM and even higher in PV patients. Their results indicated that VCAM1^+^ macrophages are able to create a niche for reactive and neoplastic erythropoiesis and may be a therapeutic target in PV (Fujiwara et al., 2017). Although the findings from other groups' studies stressed the importance of Vcam1 in island formation, across both steady and stress erythropoiesis, Vcam1 conditional KO in mice macrophages suggests that it is not a prerequisite for BM EBI formation (Wei et al., 2019). Therefore, further studies are needed to clarify the impact of Vcam1 during EBI formation in both normal erythropoiesis and erythroid hematopoietic disorders.

Emp is a quite unique adhesion molecule, which is expressed by both erythroblasts and EBI macrophages (Soni et al., 2006). Previous studies have also shown that Emp expression is similar in both Epor^+^ (EBI) and Epor^−^ (non-EBI) macrophages (Li et al., 2019). For example, *in vitro* studies have reported that the absence of Emp may lead to disordered erythropoiesis, suggesting that a direct association between EBI macrophages and erythroblasts is essential for erythroid maturation (Hanspal et al., 1998). In addition, results from *in vivo* studies indicated that *Emp*-null embryos lead to significantly impaired erythropoiesis and severe anemia, which die perinatally (*in utero* at E19.5) (Soni et al., 2006). In this mouse model, the researchers found a dramatic increase in the number of nucleated, and immature erythrocytes in the peripheral blood as well as a decrease in the number of EBIs and F4/80^+^ macrophages in the FL, suggesting that Emp is required for erythroblast enucleation and in the development of mature macrophages (Soni et al., 2006). Interestingly, a recent study showed that BM macrophages–mediated Emp expression, but not erythroblasts, is required for BM EBI formation (Wei et al., 2019). In addition, conditional KO mouse models of *Emp*, conducted to assess its cellular and postnatal contributions, revealed that *Csf1r*-Cre– or *CD169*-Cre–mediated *Emp* deletion in macrophages leads to significant reductions in populations of BM macrophages, erythroblasts, and *in vivo* island formation, whereas *Epor*-Cre–mediated deletion had no such phenotype. Overall, these findings suggested that *Emp* has a dominant role in macrophages for BM erythropoiesis (Wei et al., 2019). Furthermore, previous studies have shown that Epor expression in EBI macrophages was lower than that in erythroblasts (An et al., 2014; Li et al., 2019; Chen et al., 2020). Consequently, we hypothesized that *Epor*-Cre mouse cannot delete *Emp* expression in EBI macrophages. In addition, knocking out *Emp* in both EBI and non-EBI macrophages illustrates non-selective features (Wei et al., 2019). In conclusion, Emp is required for EBI formation, but the mechanism is still unknown. Therefore, new *Emp* mouse models with specific deletions in EBI macrophages are expected to further elucidate the role of Emp in EBI macrophages during both FL and adult BM erythropoiesis.

Macrophages also express αV integrin, an adhesion molecule whose counter-receptor intracellular adhesion molecule 4 (ICAM4) is expressed by erythroblasts. Previous studies have revealed that αV integrin has a similar expression profile between Epor^+^ and Epor^−^ macrophages (Li et al., 2019). Although its expression is relatively lower than those of other adhesion molecules, it plays pivotal roles in maintaining EBI integrity, whereas disrupting the binding of αV integrin and ICAM4 decreases EBI numbers (Mankelow et al., 2004; Lee et al., 2006). In fact, blocking ICAM-4/αV binding results in a 70% decrease in island formation. Moreover, studies have reported a marked decrease in EBIs in *ICAM-4*–null mice in the BM (Mankelow et al., 2004; Lee et al., 2006). Conversely, the steady-state *ICAM4*-null mice exhibit normal hematocrit, hemoglobin, and RBC indices, suggesting that erythropoiesis is not adversely affected in adult animals under this condition (Lee et al., 2006). Another major finding is that secretion of ICAM4S, an ICAM4 isoform in terminal erythropoiesis, dramatically increases (Lee et al., 2003). Consequently, “ICAM4S may compete with ICAM4, thereby blocking the interaction between ICAM4 and αV, which might promote detachment of reticulocytes from the islands,” thereby enabling secretion of egress into peripheral circulation. Taken together, these findings indirectly revealed the roles of αV integrin during erythropoiesis. Future studies are expected to use mouse models expressing αV integrin with specific deletions in EBI macrophage to elucidate αV integrin's role during erythropoiesis.

**Figure 2:**
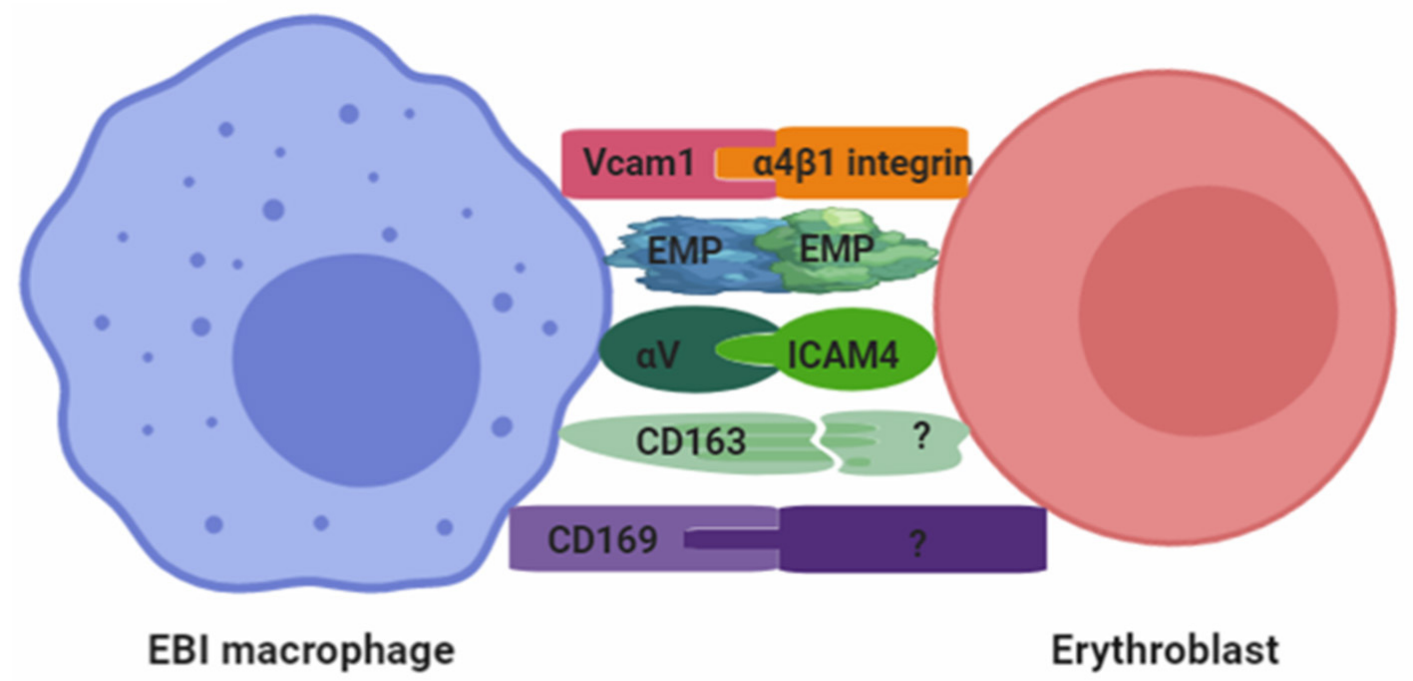
Adhesion molecules involved in the formation of EBI.

Overall, several adhesion molecules that may interact to maintain the integrity of EBI have been identified (Figure 2). It is highly likely that future studies will identify additional adhesion molecules between EBI macrophages and erythroblasts. Based on these studies, two key questions need to be addressed in the future: (1) the function of adhesion molecules expressed by EBI macrophages during erythropoiesis and (2) the dynamic of these adhesion molecules expressed by EBI macrophages associated with distinct stages of erythroblasts? This is because integrins regulate intracellular signaling (DeMali et al., 2003) and CD163 being an acute phase–regulated and signal-inducing macrophage protein, as a receptor that scavenges hemoglobin by mediating endocytosis of haptoglobin–hemoglobin complexes (Kristiansen et al., 2001). We hypothesized that some of these adhesion molecules may trigger signaling pathways that coordinate gene expression and adhesion.

### Secretion of Growth Factors Significant for Erythropoiesis by EBI Macrophages

Previous studies have shown that EBI macrophages enhance the proliferation of erythroid cells in coculture systems (Hanspal and Hanspal, 1994; Rhodes et al., 2008), although the actual mechanisms remain unclear. In addition, cytokines such as EPO, SCF, IL-3, and insulinlike growth factor 1 (IGF1) have been found to promote erythropoiesis. Study from An's group showed the expressions of I*gf1, IL-18*, and *Vegf*β, but not *EPO, SCF*, and *IL-3* in Epor^+^ EBI macrophages, suggesting that EBI macrophages may enhance erythroid proliferation by secreting these growth factors in mouse BM hematopoietic niche. Erythropoiesis-promoting cytokine *Igf1* (Li et al., 2019) is selectively expressed in EBI macrophages, and IGF1 receptor is expressed on erythroid cells across all developmental stages (Aron, 1992; Miyagawa et al., 2000). IGF1 enhances human erythropoiesis (Claustres et al., 1987; Sawada et al., 1989; Correa and Axelrad, 1991), and IGF1 often exerts its effects in an autocrine/paracrine manner traditionally (Nilsson et al., 1986; Croci et al., 2011; Chang et al., 2016; Rajbhandari et al., 2017). Based on these findings, we hypothesized that EBI macrophage-mediated local secretion of *Igf1* may contribute to erythropoiesis. To answer this question, future studies are expected to investigate *Igf1*-conditional KO in EBI macrophages. On the other hand, studies have reported the expression of IL-18 receptor in erythroid progenitors (Li et al., 2014; Yan et al., 2018), and IL-18 has been shown to promote cell proliferation in non-erythroid cells (Victor et al., 2017; Almutairi et al., 2019). However, the role of IL-18 during erythropoiesis remains unknown. Moreover, vascular endothelial growth factor A (VEGF-A) has been found to induce production of Epo under normoxic and non-anemic conditions, thereby expanding erythropoiesis (Greenwald et al., 2019). In fact, knocking out *Vegfc* in embryonic day 7.5 (E7.5) resulted in defective erythropoiesis, characterized by anemia and lack of enucleated RBCs in blood circulation (Fang et al., 2016). However, *Vegfc* loss did not disrupt generation of primitive erythroid cells or erythromyeloid progenitors in the yolk sac, although it decreased expression of α4-integrin on erythromyeloid progenitors and compromised erythromyeloid progenitors colonization of the FL. *Vegfc* KO has also been implicated in compromised distribution, maturation, and enucleation of primitive erythroblasts. In contrast, *Vegfc* deletion, from E10.5 onward, did not compromise definitive hematopoiesis in the liver and did not cause anemia in adult mice (Fang et al., 2016). Overall, these findings indicated that Vegfc is required for transition to fetal erythropoiesis. On the other hand, VEGF-B, discovered a long time ago, is thought to be a potent therapeutic agent against oxidative stress–related diseases, insulin resistance, and type 2 diabetes (Hagberg et al., 2012; Arjunan et al., 2018), however, unlike VEGF-A and VEGF-C, whose function during erythropoiesis has been extensively studied. The functions of VEGF-B as well as the associated mechanisms in erythropoiesis are poorly understood. Future studies are expected to focus on the roles of growth factors secreted by EBI macrophages, to further advance our knowledge on whether local growth factors affect erythropoiesis under both steady-state and stress conditions.

### Senescent RBC Clearance and Iron Recycling by EBI Macrophages

**Figure 3:**
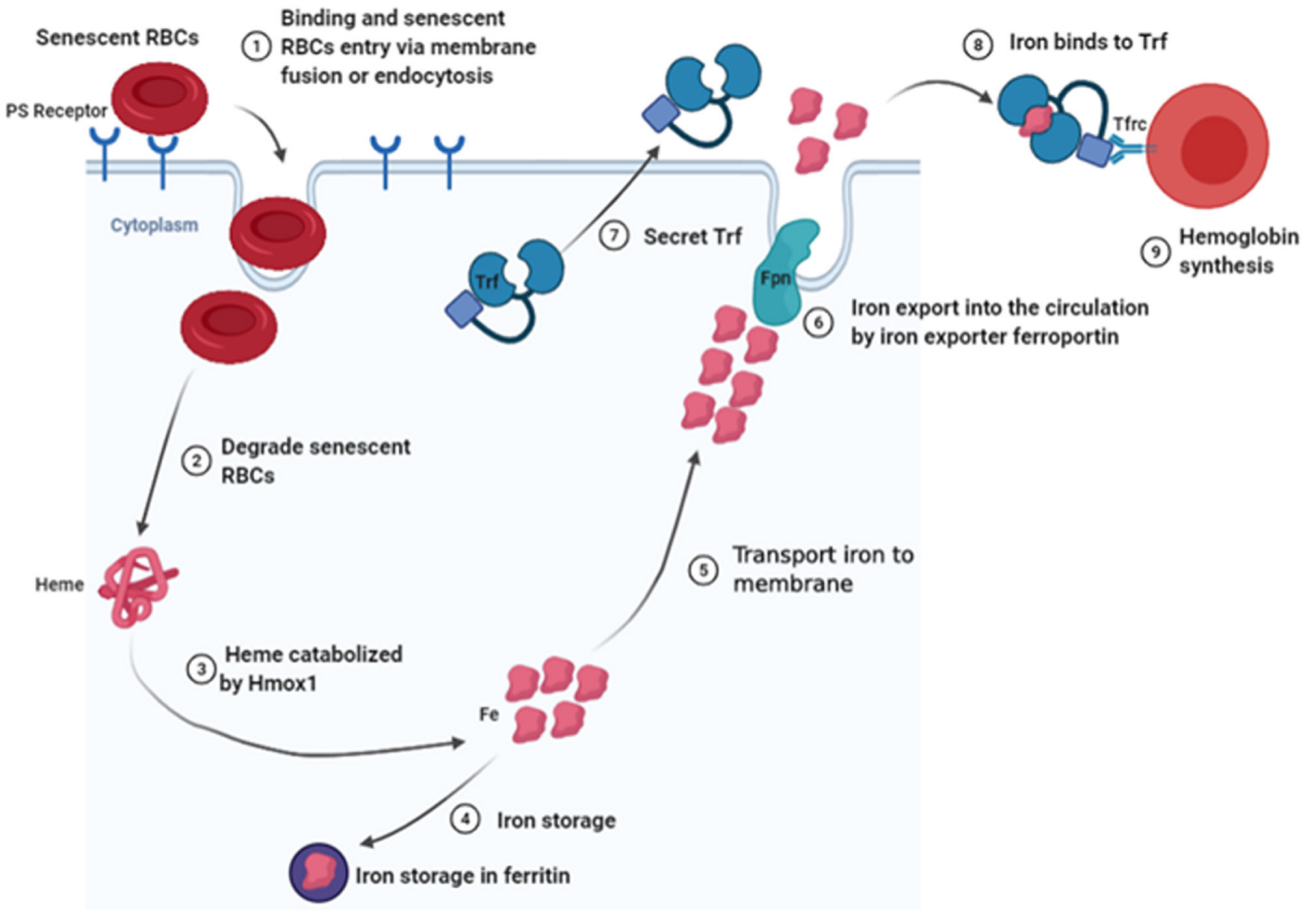
Schematic diagram of senescent RBC clearance and iron recycling by EBI macrophages in local BM microenvironment.

Generally, RBC clearance is thought to take place mainly in the spleen, where senescent RBCs are phagocytosed and degraded by splenic macrophages (Bratosin et al., 1998), and the heme is catabolized by heme oxygenase-1 (Hmox1) (Poss and Tonegawa, 1997; Kovtunovych et al., 2010). Consequently, iron for storage in ferritin is released (Leimberg et al., 2008) or exported into the circulation system by action of an iron exporter ferroportin (Fpn) (Nemeth et al., 2004). The iron subsequently binds onto transferrin (Trf) and is shuttled to the BM (Cumming, 1978; Trenor et al., 2000). Thereafter, Trf binds to Trf receptor (Tfrc), which is followed by internalization of the resulting Trf–Tfrc complex, and endosomal acidification, resulting in release of iron from Trf and transfer of iron to the cytosol for use in erythropoiesis (Muckenthaler et al., 2017). Studies have shown that Hmox1 expressed by EBI macrophages is critical for iron recycling during a steady-state erythropoiesis, with *Hmox1-*deficient mice found to exhibit low RBC sizes and hemoglobin content (Fraser et al., 2015). Based on this study, it is evident that *Hmox1* deficiency increases RBCs lifespan, possibly by attenuating macrophage-mediated RBC clearance in the spleen, liver, and BM, as well as by decreasing both macrophage numbers and Timd4 expression. Indeed, Timd4 bounds this type of cells via recognizing phosphatidylserine (PS), which is exposed by apoptotic cells and nuclei expelled by late stage of erythroid cells. This works as an “eat me” signal for phagocytes (see the detailed description in *Nuclei Engulfed and Digested by EBI Macrophages*). Other studies have reported the accumulation of iron in hepatocytes and kidneys of *Hmox1*^−/−^mice, suggesting that cells in these tissues may compensate for decreased RBC clearance by splenic macrophages, as well as the associated reduction in heme degradation (Poss and Tonegawa, 1997; Kovtunovych et al., 2010). Additionally, *Hmox1* deficiency has been found to play an important role in regulating EBI formation in the BM, which is further associated with decreased expression of Vcam1 on EBI macrophages (Fraser et al., 2015). Interestingly, Rouault's group reported that infused wild-type macrophages reside and self-renew in the liver and may rescue the hemolysis and anemia of *Hmox1*-deficient mice (Kim et al., 2018). Their findings indicated that normal EBI macrophages are critical for erythropoiesis. In fact, macrophages release iron into the bloodstream via a membrane-bound iron export protein, FPN (Lim et al., 2018), whereas hepcidin promotes the degradation of its receptor, the sole known cellular iron exporter FPN, resulting in iron retention by macrophages and reducing intestinal iron absorption (Nemeth and Ganz, 2009). Cell-specific KO of *Fpn* in intestinal epithelial cells was found to result in severe anemia (Donovan et al., 2005), whereas cell-specific KO of *Fpn* in macrophages or hepatocytes caused serum iron deficiency and mild anemia (Zhang et al., 2011, 2012). These findings confirmed that Fpn expression in the three tissues plays a crucial role in maintaining systemic iron homeostasis. Additionally, erythroid-specific *Fpn* KO mice exhibited low serum iron levels, as well as tissue iron overload and increased Fpn expression in spleen and liver, without changing hepcidin levels (Zhang et al., 2018). Furthermore, studies have described the Trf, a serum-abundant metal-binding protein, which is primarily synthesized in the liver and plays a key role in iron homeostasis and erythropoiesis (Huggenvik et al., 1989). For example, hepatocyte-specific *Trf* KO mice, which are viable and fertile, exhibit impaired erythropoiesis and altered iron metabolism, as well as iron deficiency anemia (Yu et al., 2020). In addition, *Trf* is also expressed by EBI macrophages in mouse BM, suggesting that EBI macrophages may secrete Trf in the local BM microenvironment to provide iron to erythroblasts (Li et al., 2019). To further elucidate the role of *Trf* in EBI macrophages, during erythropoiesis, future studies are expected to target macrophage-specific *Trf* KO mice. Intriguingly, RNA-seq analyses revealed the existence of an iron-recycling machinery in newly identified EBI macrophages, raising the novel concept that EBI macrophages could provide iron to the developing erythroblasts within the niche to support erythropoiesis (Figure 3) (Li et al., 2019). As iron is the most needed nutrient for erythropoiesis, it will be interesting to see whether EBI macrophages locally provide iron to the developing erythroid cells. Existence of an iron-recycling machinery in EBI macrophages strongly suggests this possibility. To understand whether BM EBI macrophages provide iron for erythropoiesis within the niche, future studies are expected to generate conditional KO mouse models with selective deletion of *Fpn* in BM EBI macrophages.

### Nuclei Engulfed and Digested by EBI Macrophages

**Figure 4:**
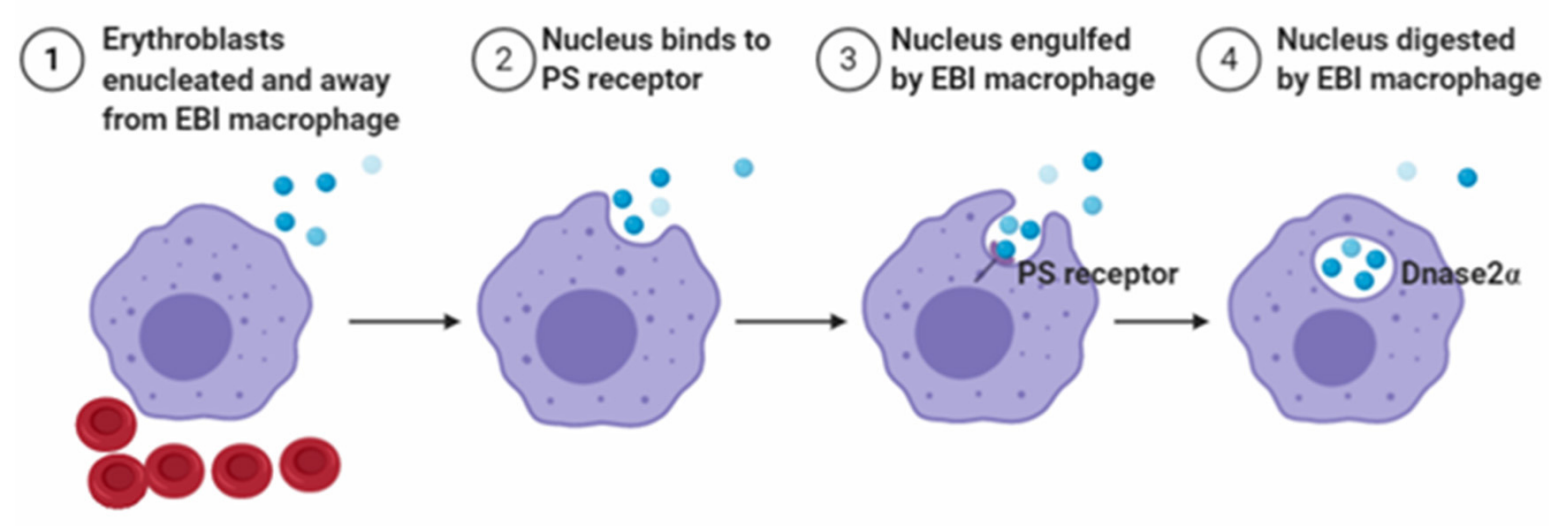
Schematic diagram of nucleus engulfed and digested by EBI macrophages.

To maintain homeostasis, EBI macrophages engulf extruded nuclei during the last step of erythroblasts maturation. In addition, the high rate of nuclei generation at steady state requires EBI macrophages to sustain a high level of phagocytic capacity. If nuclei are not swiftly engulfed, they undergo secondary necrosis and release intracellular components that activate the immune system, thereby leading to autoimmune diseases, such as systemic lupus erythematosus (Arandjelovic and Ravichandran, 2015; Toda et al., 2015). Previous studies have reported that some adhesion molecules, such as Emp and β1-integrin, are associated with engulfed extruded nuclei from erythroblasts (Lee et al., 2004; Soni et al., 2006). Functionally, these molecules effectively enable the nuclei to maintain macrophage attachments before phagocytosis. A related study reported that *Emp*-null embryos die perinatally (Soni et al., 2006) because of an increase in the number of nucleated and immature erythrocytes in peripheral blood. In addition, no EBIs were observed in the FL, and the number of F4/80^+^ macrophages was substantially reduced (Soni et al., 2006). These findings suggest the importance of Emp for erythroblast enucleation, as well as the development of mature macrophages (Soni et al., 2006). Another study demonstrated how nuclei expelled from erythroblasts. The nuclei expose PS (PtdSer) on their surface, which act as an “eat me” signal and are subsequently phagocytosed by EBI macrophages (Yoshida et al., 2005). In addition, PtdSer is exposed through activation of phospholipid scramblase with inactivation of phospholipid flippase, which are caspase-mediated events (Arandjelovic and Ravichandran, 2015; Toda et al., 2015). To recognize PtdSer, macrophages express a variety of molecules and use a sophisticated mechanism to engulf nuclei (Toda et al., 2015). Additionally, mice with mutant of milk–fat–globule EGF8 (Hanayama et al., 2002) that binds PtdSer (Yoshida et al., 2005) and inhibits phagocytosis of apoptotic cells reportedly blocked phagocytosis of extruded erythroblast nuclei, with strong evidence indicating that recognition signals for expelled nuclei are similar to those in apoptotic cells. After engulfment of the nuclei, DNase2α expressed-EBI macrophages degrade the ingested nuclear DNA (Kawane et al., 2001), and the process is outlined in Figure 4. Importantly, studies using *DNase2*α KO mice have shown the accumulation of phagocytosed nuclei in F4/80^+^ EBI macrophages, and the DNA cannot be degraded. In addition, the undegraded nuclei are toxic to macrophages, resulting in decreased numbers of F4/80^+^ EBI macrophages associated with erythroblasts, and this leads to severe anemia (Kawane et al., 2001). Moreover, Luo et al. (2016) showed that peritoneal and splenic macrophages express Epor, and Epo enhances the phagocytosis of apoptotic cells by activating nuclear receptors peroxisome proliferator-activated receptor gamma (PPARγ) and RXR. Recently, An's group found that BM and FL EBI macrophages express Epor (Li et al., 2019). In addition, the Epor^+^ macrophages also exhibited high levels of *Mertk* and *Timd4* expression, which are associated with phagocytosis. Functionally, *DNase2*α is required for DNA degradation, which also expressed high levels in Epor^+^ macrophages. Thus, it is likely that Epor^+^ macrophages may engulf nuclei extruded from erythroblasts, and Epo may also play important roles in phagocytosis of nuclei by EBI macrophages both in BM and FL. Future studies targeting KO of *Epor* in EBI macrophages are expected to ascertain whether Epo enhances phagocytosis of nuclei.

### Selective Expression of Transcription Factors by EBI Macrophages Shape Their Function

Transcription factors form the point of convergence for multiple signaling pathways within eukaryotic cells and play significant regulatory roles in the function of all cell types (Papavassiliou and Papavassiliou, 2016). To date, four selective transcription factors, expressed by EBI macrophages, have been identified in mouse BM (Li et al., 2019). These include *Klf1, Spic, Nr1h3*, and *Maf*. Bieker's group used pEKLF/GFP mice to show that ~36% of E13.5 mouse FL F4/80^+^ macrophages express EKLF (Klf1) (Xue et al., 2014). Interestingly, DNase2α and Vcam1, which significantly promote nuclei digestion and EBI formation, were found to be highly expressed in F4/80^+^Klf1/GFP^+^ than F4/80^+^Klf1/GFP^−^ macrophages. Similarly, ~31% of E14.5 mouse FL F4/80^+^macrophages were reported to express Epor^−^eGFP^+13^, which is consistent with this study. Importantly, *Klf1* KO mice were found to die *in utero* due to severe anemia, whereas residual circulating RBCs retained their nuclei. In this mouse model, FL macrophages showed altered morphology (generally round and small) and did not show extensive cytoplasmic projections of normal macrophages (Porcu et al., 2011). Additionally, the number of F4/80^+^ macrophages was dramatically decreased in E14.5 FL of *Klf1*-null mice. Interestingly, mice lacking *Klf1* also exhibited a dramatic decrease in DNase2α, whereas *Klf1* binds and activates *DNase2*α promoter, indicating that *DNase2*α is a *Klf1* target gene (Porcu et al., 2011). Importantly, *DNase2*α-null mice exhibited a severe defect in erythropoiesis and embryonic E17 of lethal anemia, evidenced by characteristic definitive circulating erythrocytes that retained their nuclei (Kawane et al., 2001). Collectively, these data demonstrated some similarities between *Klf1*-null and *DNase2*α-null phenotypes. Previous studies have also shown that Klf1 is not detectable in either FL macrophages, after several days of culture (Frontelo et al., 2007), or induced pluripotent stem cell (iPSC)–derived macrophages (Wu et al., 2018). However, inducible activation of Klf1 in iPSC-derived macrophages generated an EBI macrophage-like phenotype, which mediated an increase in production of mature, enucleated erythroid cells from umbilical cord blood CD34^+^ hematopoietic progenitor cells, and iPSCs. This enhancement is partly attributed to Klf1-induced secreted proteins, such as ANGPTL7, IL-33, and SERPINB2 (Lopez-Yrigoyen et al., 2019). Additionally, activation of Klf1 in iPSC-derived macrophages further elevated expression of other targeting adhesion molecules, including CD163, CD169, and PECAM1 (Lopez-Yrigoyen et al., 2019). Taken together, these studies indicated that Klf1 is one of the selective transcription factors that have partly shaped the function of EBI macrophages during erythropoiesis. Future studies seeking to further identify *Klf1* target genes in EBI macrophages are expected to carry out in EBI macrophage-specific *Klf1* KO mice. Previous studies have reported an elevated expression of Spic in red pulp macrophages (RPMs), a distinct splenic subset of macrophages involved in the clearing of the senescent RBCs (Kohyama et al., 2009). In fact, *Spic*^−/−^ mice were found to have a selective loss of RPMs, suggesting an important role of Spic in maintaining efficient phagocytosis of RBCs in the spleen (Kohyama et al., 2009). Additionally, Vcam1, which is highly expressed in RPMs, was virtually absent in *Spic*^−/−^ mice spleen, suggesting that this molecule may be one of *Spic*'s target genes. Moreover, Spic reportedly regulated the development of F4/80^+^VCAM1^+^ BM macrophages and that Spic expression in BM macrophages and RPM development was induced by heme, a metabolite of erythrocyte degradation (Haldar et al., 2014). Studies using *Spic*-EGFP mouse model have revealed that *Spic*-EGFP^hi^ BM macrophages were F4/80^hi^Vcam1^hi^CD11b^low^CD169^+^CD68^+^BM macrophages (Haldar et al., 2014), which is consistent with the results from surface marker profiles of EBI macrophages (Chow et al., 2013; Li et al., 2019). On the other hand, a significant reduction in CD169 expression was reported in *Spic*^−/−^ mice BM, indicating that a loss of erythropoiesis-supporting macrophages was observed (Haldar et al., 2014). Similar to ablating CD169^+^BM macrophages, *Spic*^−/−^ mice were found to exhibit reduced frequencies of erythroblasts in BM (Haldar et al., 2014). Furthermore, hemoglobin recovery from hemolytic anemia appeared to be impaired in *Spic*^−/−^mice, albeit not significantly (Haldar et al., 2014). Apart from the aforementioned molecules, previous studies have also described Nr1H3, also known as liver X receptors (LXRα and LXRβ), members of the nuclear receptor family of transcription factors that play essential roles in transcriptional control of lipid metabolism, as well as innate and adaptive immune responses. Results from studies targeting *Nr1H3* have confirmed the importance as therapeutic target for treatment of chronic inflammatory disorders (Levin et al., 2005). In addition, c-Maf has been found to play a crucial role in definitive erythropoiesis, which accompanies EBI formation in FL (Kusakabe et al., 2011), as evidenced by a significant reduction of mature erythroid compartments, but not CFU-E colony, in *c-Maf*^−/−^embryos relative to normal littermates. Interestingly, the number of erythroblasts, surrounding the macrophages in EBIs, was significantly reduced in *c-Maf*^−/−^embryos, whereas low levels of VCAM-1 were recorded in *c-Maf*^−/−^ FL macrophages (Kusakabe et al., 2011). In summary, the aforementioned selective transcription factors may collectively shape the function of EBI macrophages. To comprehensively elucidate the detailed roles of these transcription factors in EBI macrophages during steady-state conditions, as well as in response to anemic stress erythropoiesis, future studies are expected to employ KO experiments in mouse models.

### EPOR Signaling in EBI Macrophages During Steady-State Erythropoiesis and Stress Erythropoiesis

In adults, erythropoiesis is a dynamic and constant process that generates mature RBCs (Yan et al., 2017). Homeostasis of the steady-state erythropoiesis is mainly regulated by EPO, which occurs in both mouse and human BM (Wojchowski et al., 2006; Sathyanarayana et al., 2008). During this process, BM EBI macrophages mainly support the proliferation and maturation of erythroid cells (BESSIS, 1958); however, senescent erythrocytes are engulfed by RPMs in the spleen (Klei et al., 2017). Therefore, the balance between production and destruction of senescent erythrocytes is tightly regulated to maintain optimal erythrocyte concentration in the blood. Decreased number of erythrocytes results in insufficient oxygen supply into tissues, whereas excessive number of erythrocytes causes high blood viscosity, which compromises blood flow, hence reducing oxygen supply. Stress erythropoiesis maintains erythroid homeostasis when steady-state erythropoiesis is impaired (Paulson et al., 2020). Stress erythropoiesis is mainly modulated by splenic erythropoiesis, which are distinct from BM steady-state erythropoiesis (Liao et al., 2018b). During stress condition, spleen RPMs secreted C-C motif chemokine ligand 2, which then recruit monocytes into the spleen, and these monocytes develop into macrophages that form EBIs (Liao et al., 2018b). These studies indicated that BM and splenic macrophages have distinct roles in supporting erythropoiesis. Studies will be needed to further investigate the distinct roles of BM and splenic macrophages in regulating the generation of erythrocytes by steady and stress erythropoiesis.

Although macrophages are primarily involved in immune functions, prevailing evidence indicated that they have additional roles, including regulating the hematopoietic microenvironment, influencing metabolism, mediating tissue repair, and overseeing the maturation of embryonic tissue (Bian et al., 2020). Frenette's group found that specific depletion of CD169^+^ macrophages markedly decreases the number of erythroblasts in the BM but does not result in overt anemia under homeostatic conditions, probably because of concomitant alterations in RBC clearance (Chow et al., 2013). However, depletion of CD169^+^ macrophages significantly impaired erythroid recovery from hemolytic anemia, caused acute blood loss and myeloablation (Chow et al., 2013). Interestingly, previous studies showed that stress erythropoiesis is dependent on BMP4, growth differentiation factor 15, SCF, Hedgehog, and EPO (Harandi et al., 2010; Chow et al., 2013; Hao et al., 2019; Chen et al., 2020). The significance of BMP4 has been well-confirmed by Paulson's group, showing that BMP4 signal pathway dysfunction significantly impairs the development of SEPs, while delaying peripheral erythroid recovery in response to acute hemolytic anemia (Lenox et al., 2005; Harandi et al., 2010). Frenette's group found that depletion of macrophages by clodronate liposome or depletion of CD169^+^ macrophages after BMT impairs BMP4-mediated stress erythropoiesis, which is consistent with the finding of Paulson's group (Harandi et al., 2010) and suggests that BMP4 might be derived from splenic RPMs. Taken together, senescent RBCs are cleared by RPMs in general; however, BMP4-producing RPMs, monocyte-derived macrophages, and Epor^+^ macrophages may work in concert to mediate stress erythropoiesis (Chow et al., 2013; Li et al., 2019; Chen et al., 2020). However, there is no conclusion whether these macrophages are the same or distinct subpopulations of macrophages or they have some cross. Studying the heterogeneity of FL, BM and splenic macrophages during erythropoiesis would be the key to developing new mechanisms to better understand erythropoiesis both in steady and stress state.

It has also been reported that macrophages contribute to increased erythroid activity following administration of human recombinant EPO (Ramos et al., 2013; Wang et al., 2018; Li et al., 2019). Rivella's group reported that clodronate administration impairs erythroid expansion in the spleen, as well as RBC and reticulocyte production in response to EPO supplementation, suggesting the potential contribution of macrophages in response to EPO administration (Ramos et al., 2013). Huang's group demonstrated that activation of Epor signaling increases both erythrocytes and macrophages in mice models of primary and secondary erythrocytosis (Wang et al., 2018). To explore the mechanisms, the authors showed that most of macrophages do not express Epor; however, a tight interaction between macrophages and Epor-expressing erythroid cells was found. The authors thought that this may affect the judgment of Epor expression by macrophages, because they cannot exclude the possibility that a subpopulation of macrophages, which are the EBI macrophages binding to erythroblasts, thereby expresses Epor on their cell surface (Wang et al., 2018). An's group further pointed that EBI macrophages do express Epor via *in vitro* and *in vivo* experiments (Li et al., 2019). Significantly, EPO enhances the EBI formation via increasing the production of erythroblasts and EBI macrophages, as well as the expression of adhesion molecules Vcam1 and CD163 in mouse BM (Li et al., 2019). Stress erythropoiesis is best understood in mice where it is extramedullary occurring primarily in the spleen (Liao et al., 2018a,b). EPO administration changes spleen from a non-erythropoietic organ into a fully erythropoietic one as demonstrated by the dramatic increases in both erythroblasts and EBIs, accompanied by the increase in Epor^+^ macrophages. Importantly, Epor^+^ macrophages form stress splenic EBIs, demonstrating the role of Epor^+^ macrophages in splenic stress erythropoiesis (Li et al., 2019). Anemic stress induces the rapid proliferation of SEPs and differentiation after increase in EPO levels (Liao et al., 2018b). Paulson's group also confirmed the expression of Epor in splenic macrophages using macrophages-specific KO STAT5 mice and found that these Epor^+^ splenic macrophages could support the proliferation and differentiation of SEPs (Chen et al., 2020). However, previous studies showed that developing erythroid cells, ranging from CFU-E to terminal erythroid cells, are found to be associated with EBI macrophages. These studies suggested that EBI macrophages support proliferation and differentiation of CFU-E and terminal erythroid maturation in general. The very recent and exciting work reported by Paulson's group indicated that the differentiation of immature SEPs is also regulated by splenic Epor^+^ macrophages, which extends the stages of erythroid cells regulated by EBI macrophages (Chen et al., 2020). They reported that Epo promotes SEP transition from proliferation to differentiation by acting on macrophages in the splenic niche (Chen et al., 2020). During the process of proliferation of SEPs, macrophages produce Wnt ligands, which promote proliferation and inhibit differentiation of SEPs. Specific deletion of Stat5 in macrophages significantly disturbed Epo/stat5-dependent signal transduction and inhibited the production of bioactive lipid mediators in macrophages (Chen et al., 2020). Decreased production of prostaglandin J_2_ inhibited PPARγ-dependent repression of Wnt expression, whereas decreased production of prostaglandin E_2_ inhibited the differentiation of SEPs (Chen et al., 2020). In addition, human cord blood–derived macrophages express EPOR, but late-stage erythroblasts do not relay on EPO to survive and mature. Coculturing these macrophages with erythroblasts, An's group found that EPO enhances EBI formation (Li et al., 2019). These data indicated that Epor signaling pathway in EBI macrophages contributes to steady-state erythropoiesis and stress erythropoiesis. EPO regulates erythroid development, and Epo/Epor-mediated signaling transduction in macrophages regulates erythropoiesis (Wang et al., 2018; Li et al., 2019; Paulson, 2019; Chen et al., 2020). Therefore, a novel concept is that Epo simultaneously acts on both erythroid cells and EBI macrophages in the niche to ensure efficient erythropoiesis. Consequently, the potential knowledge for Epo to regulate the function of EBI macrophages and erythroblasts opens a new frontier for future research into the coordinated regulation of differentiation of erythroblasts and their niche to ensure efficient erythropoiesis (Paulson, 2019).

In conclusion, transcriptomic data from F4/80^+^Epor^−^eGFP^+^ and F4/80^+^Epor^−^eGFP^−^ macrophages provide new insights into potential mechanisms by which EBI macrophages support erythropoiesis. Data show that inhibition of Epo/stat5 signaling in macrophages impairs stress erythropoiesis in the splenic niche. Given the fact that EPO/EPOR is essential for the survival of erythroid progenitors and early-stage erythroblasts (Koury and Bondurant, 1990), while EBI macrophages do not require EPO/EPOR for survival, these suggest the downstream effectors of EPO/EPOR-mediated signal pathways in EBI macrophages are different from those in erythroid cells. In erythroid cells, EPO administration leads to upregulation of antiapoptotic genes (Sathyanarayana et al., 2008). Interestingly, it has been reported that EPO administration led to increase in phagocytosis activity via upregulation of PPARγ in both peritoneal macrophages and spleen niche macrophages (Luo et al., 2016; Chen et al., 2020). Study also reported that EPO administration resulted in increased EBI macrophage numbers and the surface expression of adhesion molecule Vcam1 (Li et al., 2019). Furthermore, it is needed to uncover additional EPO/EPOR-mediated molecules/pathways in the EBI macrophages through RNA-seq analyses. All the specific molecules associated with EBI macrophages and KO hematopoietic phenotype of these proteins reported so far are listed in Table 1. Several other differentially expressed genes between F4/80^+^Epor^−^eGFP^+^ and F4/80^+^Epor^−^eGFP^−^ macrophages such as *Apoe, C1qa, C1qb, C1qc, Cd5l, Mrc1, myb, Mmp14*, and *Slc48a1* (Li et al., 2019) are also highly or selectively expressed in EBI macrophages; however, the roles of these genes during erythropoiesis are still unknown. In the future, further studies of these unknown molecules expressed on EBI macrophages will further advance the field of EBI macrophages during erythropoiesis.

**Table 1:** List of markers of EBI macrophages in mice and human and potential hematopoietic phenotype of gene deletion mice models.

	**Mice**	**Human**	**Hematopoietic phenotype of gene deletion in mouse**
EPOR	+	+	Stat5^−/−^ in macrophages impaired stress erythropoiesis and Epor^−/−^ in macrophages impaired apoptotic cells clearance.
F4/80	+	+	No report
CD11B	+/-	+	No report
CD45	+	+	No report
Ly6C	~69%+	-	No report
Ly6G	-	-	-
VCAM1	+	+	Vcam1^−/−^ in adult BM showed no hematopoietic phenotypes.
EMP	+	+	EMP^−/−^ mice die *in utero* at E19.5 and EMP^−/−^ in macrophages in adult BM impaired EBI formation.
CD163	~ 35%+	+	No report
CD169	+	+	Deletion of CD169^+^ macrophages impaired stress erythropoiesis.
αV	+	+	EBIs are markedly decreased in ICAM-4^−/−^mice in the BM.
ER-HR3	+	+	No report
CD206	~ 15%+	+	No report
TIMD4	+	+	TIMD4 is a phosphatidylserine receptor for the engulfment of apoptotic cells and nucleus.
MERTK	+	+	Axl^−/−^Mertk^−/−^ mice impaired erythropoiesis in bone marrow and expanded splenic erythropoiesis.
AXL	+	+	Axl^−/−^Mertk^−/−^ mice impaired erythropoiesis in bone marrow and expanded splenic erythropoiesis.
DNASE2α	+	+	DNASE2α^−/−^ mice die in embryonic E17 because of lethal anemia.
HMOX1	+	+	*Hmox1^−/−^*mice exhibits decreased in RBCs size and hemoglobin content.
FPN	+	+	Fpn^−/−^ in macrophages resulted in serum iron deficiency and mild anemia.
TRF	+	+	Hepatocyte-specific Trf knockout mice resulted in iron deficiency anemia.
IGF1	+	+	IGF1 enhances human erythropoiesis.
IL18	+	+	IL18 promotes NK cell proliferation.
VEGFβ	+	+	No report
KLF1	+	+	Klf1^−/−^ die *in utero* due to severe anemia.
SPIC	+	+	Spic^−/−^ mice exhibit reduced frequencies of pro-E in BM and impaired stress erythropoiesis.
NR1H3	+	+	No report
MAF	+	+	c-Maf^−/−^ mice impaired FL erythropoiesis.

*CD, Cluster of differentiation*.

## EBI Macrophages Drive Erythroid Hematopoietic Disorders

Studies show that EBI macrophages function as “nurse cells” providing iron and growth factors during erythropoiesis. The EBI macrophages also engulf and digest extruded erythroid nuclei. EBIs have recently been found to play clinically important roles in erythroid hematopoietic disorders, such as β-thalassemia, and PV (Chow et al., 2013; Ramos et al., 2013). β-Thalassemia and PV are genetic disorders, which mainly affect erythropoiesis due to persistent high erythropoietic activity (Crielaard and Rivella, 2014). Most patients with PV present high hemoglobin levels because of excessive RBC synthesis, whereas patients with β-thalassemia exhibit impairment in β-globin synthesis, leading to defective RBCs and anemia. In 2013, Ramos et al. reported that macrophages facilitate recovery from induced anemia and prevent the pathological progression of PV and β-thalassemia by modulating erythroid proliferation and differentiation (Ramos et al., 2013). Importantly, macrophage depletion normalized the erythroid compartment in a JAK2(V617F)-driven mouse model of PV, suggesting that erythropoiesis in PV remains under the control of macrophages in the BM and splenic hematopoietic niche (Chow et al., 2013). This further shows the contribution of EBI macrophages in the pathological progression of PV and β-thalassemia. Therefore, EBI macrophages may be a novel target of therapeutic interventions. CSF1R inhibitor and anti-CD47 antibodies or anti-SIRPα antibodies have been applied for solid tumors and some hematologic malignancies such as acute myeloid leukemia and myelodysplastic syndrome (MDS) (Weiskopf, 2017). However, there are no reports about these therapies in PV, given the fact that anemia is one key adverse effect of these therapies. Therefore, we speculate that CSF1R inhibitor, anti-CD47 antibodies or anti-SIRPα antibodies, may be a novel therapy option for PV via targeting macrophages. Clinical studies are worthy to confirm this hypothesis. In addition, further studies are also needed to explore the function of EBI macrophages in hematological diseases such as ineffective erythropoiesis of thalassemia and the expanded erythron in PV. A cross between β-thalassemia mice with *Epor-*eGFPcre mice may provide a mouse model which could allow further investigation into the contribution of EBI macrophages to the pathophysiology of erythropoietic disorders β-thalassemia, given the fact that *Epor-*eGFPcre mice will elicit both erythroid cell–autonomous and EBI effects. A cross between JAK2 ^V617F/+^mice with *Spic*-EYFPCre or *Csf1r*-Cre or *CD169*-Cre mice would allow further investigation into the specific contribution of EBI macrophages on the pathophysiology of PV, as *Spic, Csf1r*, and *CD169* are expressed by EBI macrophages but not erythroid cells (An et al., 2014; Li et al., 2019). Of these three murine Cre lines, *Spic*-EYFPCre mice may play more significant roles in exploring the contributions of EBI macrophages in PV than the other two Cre lines. Because *Spic* is selectively expressed by EBI macrophages, however, *Csf1r* and *CD169* are both expressed by EBI macrophages and non-EBI macrophages (Li et al., 2019).

Sickle cell disease (SCD) is an autosomal recessive genetic RBC disorder that is widely distributed globally (Lombardi et al., 2019). Hemolytic disease is a feature of SCD, which presents with high amount of hemoglobin and heme in the peripheral circulation (Kato et al., 2017), heme-iron loading in reticuloendothelial system macrophages, and chronic inflammation (Vinchi et al., 2016). In this condition, Vinchi et al. reported that heme and iron could polarize macrophage toward an M1-like proinflammatory phenotype *in vitro* and *in vivo* (Vinchi et al., 2016). Heme and iron increased the expression of MHCII, tumor necrosis factor α, CD86, CD14, IL-6, and IL-1β and decreased the expression of CD206, IL-10 and agrinase-1 in macrophages. A similar macrophages phenotype was seen in SCD mice (Vinchi et al., 2016). Importantly, hemopexin, a heme scavenger, has been found to attenuate the inflammatory phenotype of macrophages in SCD mice (Vinchi et al., 2016). These data suggest that macrophages contribute to the pathogenesis of SCD, whereas hemopexin could counteract the heme-driven macrophage-mediated inflammation, which is a featured pathophysiologic consequence of SCD. In addition, inflammatory disorders may induce anemia, and these conditions are commonly considered as the activation of innate immune system, which includes the monocyte/macrophage lineage (Akilesh et al., 2019). Based on this, Akilesh et al. reported that inflammation caused anemia via inducing inflammatory Ly6C^hi^ monocytes differentiated into inflammatory hemophagocytes (iHPCs) (Akilesh et al., 2019). These iHPCs shared the expression of key transcription factor Spic with RPMs but showed increased phagocytic uptake of RBCs. They also found that the development and differentiation of iHPCs from inflammatory Ly6C^hi^ required cell-intrinsic toll-like receptor 7 signaling (Akilesh et al., 2019). Significantly, deletion of monocytes caused a severe reduction in iHPC numbers and rescued this inflammatory anemia (Akilesh et al., 2019). Spic is a selective transcription factor expressed by EBI macrophages, which suggest these iHPCs also express Epor, whereas Epo/Epor signaling pathway in macrophages has been reported to have anti-inflammatory functions (Luo et al., 2016; Li et al., 2019; Paulson, 2019). Taken together, SCD is an inflammatory anemia at some extent, and macrophages remolding or reactivation of Epo/Epor signaling in macrophages may be another treatment option for SCD.

Erythroid hematopoietic disorders include PV, β-thalassemia, SCD, congenital dyserythropoietic anemia (Wickramasinghe and Wood, 2005; Heimpel et al., 2006; Schwarz et al., 2009), Diamond-Blackfan anemia (Diamond et al., 1976; Lipton and Ellis, 2009; Ashley et al., 2020), malarial anemia (Chang et al., 2004; Casals-Pascual et al., 2006; Haldar and Mohandas, 2009), and MDS (Ebert et al., 2008; Nimer, 2008). Further studies should be conducted to delineate the roles of EBI macrophages during erythroid hematopoietic disorders. The role of macrophages, especially EBI macrophages in disease progression and pathogenesis, is not well-defined; however, it still represents novel targets of therapeutic interventions. In the future, exploring novel EBI macrophage-specific targeting strategies will further advance the field of erythroid hematopoietic disorders.

## Future Perspectives

EBI macrophages were discovered more than 60 years ago and first identified and characterized in An's laboratory. Transcriptome data of EBI macrophages generated in An's laboratory lay the foundation and provide new insights into the potential mechanisms by which they support erythropoiesis. This study also leads to a better understanding of the pathophysiology of erythropoietic disorders such as ineffective erythropoiesis of β-thalassemia, expanded erythron in PV, and inflammatory conditions of SCD. Depletion of macrophages in murine models of these diseases improved erythropoiesis, showing that EBI macrophages are novel targets for therapeutic intervention. EBI macrophages express Epor, and Epo/Epor signaling pathway regulates stress erythropoiesis in EBI macrophages. These studies indicate that signaling by EBI macrophages affects disease progression and pathology, which must be taken into account when developing new treatments of anemia and PV. Several questions remain unanswered such as (1) do EBI macrophages produce iron locally to the developing erythroid cells? The findings that molecules involved in iron recycle such as PS receptor Timd4, tyrosine kinase Mertk, Axl, Hmox1, iron exporter Fpn, and iron transporter Trf are abundant in EBI macrophages strongly suggest that EBI macrophages may provide iron locally to support erythropoiesis. (2) Does local secretion of igf1 by EBI macrophages contribute to erythropoiesis? Igf1 is expressed in EBI macrophages but not non-EBI macrophages, which enhance erythropoiesis. (3) EBI macrophages engulf nuclei extruded from erythroblasts. Does Epo enhance engulfment of nuclei by EBI macrophages? If so, what are the underlying molecular pathways? (4) As EBI macrophages express Epor, what are the additional Epo/Epor-mediated molecules/pathways in the EBI macrophages? (5) To what extent are EBI macrophages heterogonous? And what are the specific functions of each subtype in erythropoiesis? (6) How to treat erythroid hematopoietic disorders by targeting EBI macrophages? In conclusion, EBI macrophages shape normal erythropoiesis and drive erythroid hematological diseases. By developing protocols for isolation of EBI macrophages and creation of an RNA-seq database, An's group have generated foundation knowledge for future studies to expand our understanding on the roles of EBI macrophages both in normal conditions and in erythroid-associated hematological diseases.

## Author Contributions

WL wrote the manuscript, collected the related literature, and finished the figures and tables. RG, YS, and ZJ revised and edited the manuscript. All authors approved the final manuscript.

## Conflict of Interest

The authors declare that the research was conducted in the absence of any commercial or financial relationships that could be construed as a potential conflict of interest.

## References

[B1] Akilesh H. M. Buechler M. B. Duggan J. M. Hahn W. O. Matta B. Sun X.. (2019). Chronic TLR7 and TLR9 signaling drives anemia via differentiation of specialized hemophagocytes. Science 363:eaao5213. 10.1126/science.aao5213 30630901 PMC6413693

[B2] Almutairi S. M. Ali A. K. He W. Yang D. S. Ghorbani P. Wang L.. (2019). Interleukin-18 up-regulates amino acid transporters and facilitates amino acid–induced mTORC1 activation in natural killer cells. J. Biol. Chem. 294, 4644–4655. 10.1074/jbc.RA118.005892 30696773 PMC6433059

[B3] An X. Schulz V. P. Li J. Wu K. Liu J. Xue F.. (2014). Global transcriptome analyses of human and murine terminal erythroid differentiation. Blood 123, 3466–3477. 10.1182/blood-2014-01-548305 24637361 PMC4041167

[B4] Arandjelovic S. Ravichandran K. S. (2015). Phagocytosis of apoptotic cells in homeostasis. Nat. Immunol. 16, 907–917. 10.1038/ni.3253 26287597 PMC4826466

[B5] Arjunan P. Lin X. Tang Z. Du Y. Kumar A. Liu L.. (2018). VEGF-B is a potent antioxidant. Proc. Natl. Acad. Sci. U.S.A. 115, 10351–10356. 10.1073/pnas.1801379115 30249667 PMC6187152

[B6] Aron D. C. (1992). Insulin-like growth factor I and erythropoiesis. Biofactors 3, 211–216. 1376602

[B7] Ashley R. J. Yan H. Wang N. Hale J. Dulmovits B. M. Papoin J.. (2020). Steroid resistance in diamond blackfan anemia associates with p57Kip2 dysregulation in erythroid progenitors. J. Clin. Invest. 130, 2097–2110. 10.1172/JCI132284 31961825 PMC7108903

[B8] Avecilla S. T. Hattori K. Heissig B. Tejada R. Liao F. Shido K.. (2004). Chemokine-mediated interaction of hematopoietic progenitors with the bone marrow vascular niche is required for thrombopoiesis. Nat. Med. 10, 64–71. 10.1038/nm973 14702636

[B9] BESSIS M. (1958). L'ílot érythroblastique, unité fonctionnelle de la moelle osseuse. Rev. Hematol. 13, 8–11. 13555228

[B10] Bian Z. Gong Y. Huang T. Lee C. Z. W. Bian L. Bai Z.. (2020). Deciphering human macrophage development at single-cell resolution. Nature 582, 571–576. 10.1038/s41586-020-2316-7 32499656

[B11] Bratosin D. Mazurier J. Tissier J. P. Estaquier J. Huart J. J. Ameisen J. C.. (1998). Cellular and molecular mechanisms of senescent erythrocyte phagocytosis by macrophages. A review. Biochimie 80, 173–195. 10.1016/S0300-9084(98)80024-2 9587675

[B12] Casals-Pascual C. Kai O. Cheung J. O. P. Williams S. Lowe B. Nyanoti M.. (2006). Suppression of erythropoiesis in malarial anemia is associated with hemozoin *in vitro* and *in vivo*. Blood 108, 2569–2577. 10.1182/blood-2006-05-018697 16804108

[B13] Chang H. R. Kim H. J. Xu X. Ferrante A. W. (2016). Macrophage and adipocyte IGF1 maintain adipose tissue homeostasis during metabolic stresses. Obesity 24, 172–183. 10.1002/oby.21354 26663512 PMC4793714

[B14] Chang K. H. Tam M. Stevenson M. M. (2004). Inappropriately low reticulocytosis in severe malarial anemia correlates with suppression in the development of late erythroid precursors. Blood 103, 3727–3735. 10.1182/blood-2003-08-2887 14739226

[B15] Chasis J. A. Mohandas N. (2008). Erythroblastic islands: Niches for erythropoiesis. Blood 112, 470–478. 10.1182/blood-2008-03-077883 18650462 PMC2481536

[B16] Chen Y. Xiang J. Qian F. Diwakar B. T. Ruan B. Hao S.. (2020). Epo receptor signaling in macrophages alters the splenic niche to promote erythroid differentiation. Blood 136, 235–246. 10.1182/blood.2019003480 32350523 PMC7357191

[B17] Chow A. Huggins M. Ahmed J. Hashimoto D. Lucas D. Kunisaki Y.. (2013). CD169 + macrophages provide a niche promoting erythropoiesis under homeostasis and stress. Nat. Med. 19, 429–436. 10.1038/nm.3057 23502962 PMC3983996

[B18] Claustres M. Chatelain P. Sultan C. (1987). Insulin-like growth factor i stimulates human erythroid colony formation in vitro. J. Clin. Endocrinol. Metab. 65, 78–82. 10.1210/jcem-65-1-78 3584401

[B19] Correa P. N. Axelrad A. A. (1991). Production of erythropoietic bursts by progenitor cells from adult human peripheral blood in an improved serum-free medium: role of insulinlike growth factor 1. Blood 78, 2823–2833. 10.1182/blood.v78.11.2823.2823 1954373

[B20] Crane G. M. Jeffery E. Morrison S. J. (2017). Adult haematopoietic stem cell niches. Nat. Rev. Immunol. 17, 573–590. 10.1038/nri.2017.53 28604734

[B21] Crielaard B. J. Rivella S. (2014). β-Thalassemia and Polycythemia vera: targeting chronic stress erythropoiesis. Int. J. Biochem. Cell Biol. 51, 89–92. 10.1016/j.biocel.2014.03.029 24718374 PMC4083841

[B22] Croci L. Barili V. Chia D. Massimino L. Van Vugt R. Masserdotti G.. (2011). Local insulin-like growth factor i expression is essential for Purkinje neuron survival at birth. Cell Death Differ. 18, 48–59. 10.1038/cdd.2010.78 20596079 PMC3131878

[B23] Crocker P. Werb Z. Gordon S. Bainton D. (1990). Ultrastructural localization of a macrophage-restricted sialic acid binding hemagglutinin, SER, in macrophage-hematopoietic cell clusters. Blood 76, 1131–1138. 10.1182/blood.v76.6.1131.bloodjournal7661131 2205308

[B24] Crocker P. R. Gordon S. (1986). Properties and distribution of a lectin-like hemagglutinin differentially expressed by murine stromal tissue macrophages. J. Exp. Med. 164, 1862–1875. 10.1084/jem.164.6.1862 3783087 PMC2188478

[B25] Crocker P. R. Gordon S. (1989). Mouse macrophage hemagglutinin (sheep erythrocyte receptor) with specificity for sialylated glycoconjugates characterized by a monoclonal antibody. J. Exp. Med. 169, 1333–1346. 10.1084/jem.169.4.1333 2926328 PMC2189241

[B26] Cumming R. L. C. (1978). Disorders of iron metabolism. Practitioner 221, 184–192. 10.1056/nejm199912233412607 724612

[B27] DeMali K. A. Wennerberg K. Burridge K. (2003). Integrin signaling to the actin cytoskeleton. Curr. Opin. Cell Biol. 15, 572–582. 10.1016/S0955-0674(03)00109-1 14519392

[B28] Diamond L. K. Wang W. C. Alter B. P. (1976). Congenital hypoplastic anemia. Adv. Pediatr. 22, 349–378. 10.1007/978-3-540-29676-8_8325 773132

[B29] Donovan A. Lima C. A. Pinkus J. L. Pinkus G. S. Zon L. I. Robine S.. (2005). The iron exporter ferroportin/Slc40a1 is essential for iron homeostasis. Cell Metab. 1, 191–200. 10.1016/j.cmet.2005.01.003 16054062

[B30] Dzierzak E. Philipsen S. (2013). Erythropoiesis: development and differentiation. Cold Spring Harb. Perspect. Med. 3:a011601. 10.1101/cshperspect.a011601 23545573 PMC3684002

[B31] Ebert B. L. Galili N. Tamayo P. Bosco J. Mak R. Pretz J.. (2008). An erythroid differentiation signature predicts response to lenalidomide in myelodysplastic syndrome. PLoS Med. 5, 0312–0322. 10.1371/journal.pmed.0050035 18271621 PMC2235894

[B32] Fabriek B. O. Polfliet M. M. J. Vloet R. P. M. Van Der Schors R. C. Ligtenberg A. J. M. Weaver L. K.. (2007). The macrophage CD163 surface glycoprotein is an erythroblast adhesion receptor. Blood 109, 5223–5229. 10.1182/blood-2006-08-036467 17353345

[B33] Fang S. Nurmi H. Heinolainen K. Chen S. Salminen E. Saharinen P.. (2016). Critical requirement of VEGF-C in transition to fetal erythropoiesis. Blood 128, 710–720. 10.1182/blood-2015-12-687970 27343251

[B34] Fraser S. T. Midwinter R. G. Coupland L. A. Kong S. Berger B. S. Yeo J. H.. (2015). Heme oxygenase-1 deficiency alters erythroblastic Island formation, steady-state erythropoiesis and red blood cell lifespan in mice. Haematologica 100, 601–610. 10.3324/haematol.2014.116368 25682599 PMC4420209

[B35] Frontelo P. Manwani D. Galdass M. Karsunky H. Lohmann F. Gallagher P. G.. (2007). Novel role for EKLF in megakaryocyte lineage commitment. Blood 110, 3871–3880. 10.1182/blood-2007-03-082065 17715392 PMC2190608

[B36] Fujiwara H. Nishimura H. Irei I. Akiyama T. Hamazaki S. Wada H.. (2017). Human bone marrow VCAM-1 + macrophages provide a niche for reactive and neoplastic erythropoiesis. Kawasaki Med. J. 43, 79–93. 10.11482/KMJ-E43(2)79

[B37] Greenwald A. C. Licht T. Kumar S. Oladipupo S. S. Iyer S. Grunewald M.. (2019). VEGF expands erythropoiesis via hypoxia-independent induction of erythropoietin in noncanonical perivascular stromal cells. J. Exp. Med. 216, 215–230. 10.1084/jem.20180752 30545903 PMC6314526

[B38] Hagberg C. E. Mehlem A. Falkevall A. Muhl L. Fam B. C. Ortsäter H.. (2012). Targeting VEGF-B as a novel treatment for insulin resistance and type 2 diabetes. Nature 490, 426–430. 10.1038/nature11464 23023133

[B39] Haldar K. Mohandas N. (2009). Malaria, erythrocytic infection, and anemia. Hematology Am. Soc. Hematol. Educ. Program 2009, 87–93. 10.1182/asheducation-2009.1.87 PMC2933134 20008186

[B40] Haldar M. Kohyama M. So A. Y. L. Kc W. Wu X. Briseño C. G.. (2014). Heme-mediated SPI-C induction promotes monocyte differentiation into iron-recycling macrophages. Cell 156, 1223–1234. 10.1016/j.cell.2014.01.069 24630724 PMC4010949

[B41] Hanayama R. Tanaka M. Miwa K. Shinohara A. Iwamatsu A. Nagata S. (2002). Identification of a factor that links apoptotic cells to phagocytes. Nature 417, 182–187. 10.1038/417182a 12000961

[B42] Hanspal M. Hanspal J. (1994). The association of erythroblasts with macrophages promotes erythroid proliferation and maturation: a 30-kD heparin-binding protein is involved in this contact. Blood 84, 3494–3504. 10.1182/blood.v84.10.3494.bloodjournal84103494 7949103

[B43] Hanspal M. Smockova Y. Uong Q. (1998). Molecular identification and functional characterization of a novel protein that mediates the attachment of erythroblasts to macrophages. Blood 92, 2940–2950. 10.1182/blood.v92.8.2940 9763581

[B44] Hao S. Xiang J. Wu D. C. Fraser J. W. Ruan B. Cai J.. (2019). Gdf15 regulates murine stress erythroid progenitor proliferation and the development of the stress erythropoiesis niche. Blood Adv. 3, 2205–2217. 10.1182/bloodadvances.2019000375 31324641 PMC6650738

[B45] Harandi O. F. Hedge S. Wu D. C. Mckeone D. Paulson R. F. (2010). Murine erythroid short-term radioprotection requires a BMP4-dependent, self-renewing population of stress erythroid progenitors. J. Clin. Invest. 120, 4507–4519. 10.1172/JCI41291 21060151 PMC2993581

[B46] Hartnell A. Steel J. Turley H. Jones M. Jackson D. G. Crocker P. R. (2001). Characterization of human sialoadhesin, a sialic acid binding receptor expressed by resident and inflammatory macrophage populations. Blood 97, 288–296. 10.1182/blood.V97.1.288 11133773

[B47] He H. Xu J. Warren C. M. Duan D. Li X. Wu L.. (2012). Endothelial cells provide an instructive niche for the differentiation and functional polarization of M2-like macrophages. Blood 120, 3152–3162. 10.1182/blood-2012-04-422758 22919031 PMC3471522

[B48] Heideveld E. Hampton-O'neil L. A. Cross S. J. van Alphen F. P. J. van den Biggelaar M. Toye A. M.. (2018). Glucocorticoids induce differentiation of monocytes towards macrophages that share functional and phenotypical aspects with erythroblastic Island macrophages. Haematologica 103, 395–405. 10.3324/haematol.2017.179341 29284682 PMC5830394

[B49] Heimpel H. Schwarz K. Ebnöther M. Goede J. S. Heydrich D. Kamp T.. (2006). Congenital dyserythropoietic anemia type I (CDAI): molecular genetics, clinical appearance, and prognosis based on long-term observation. Blood 107, 334–340. 10.1182/blood-2005-01-0421 16141353

[B50] Huang Y. Hale J. Wang Y. Li W. Zhang S. Zhang J.. (2018). SF3B1 deficiency impairs human erythropoiesis via activation of p53 pathway: implications for understanding of ineffective erythropoiesis in MDS. J. Hematol. Oncol. 11:19. 10.1186/s13045-018-0558-8 29433555 PMC5810112

[B51] Huggenvik J. Craven C. Idzerda R. Bernstein S. Kaplan J. McKnight G. (1989). A splicing defect in the mouse transferrin gene leads to congenital atransferrinemia. Blood 74, 482–486. 10.1182/blood.v74.1.482.bloodjournal741482 2752125

[B52] Jacobsen R. N. Forristal C. E. Raggatt L. J. Nowlan B. Barbier V. Kaur S.. (2014). Mobilization with granulocyte colony-stimulating factor blocks medullar erythropoiesis by depleting F4/80+VCAM1+CD169+ER-HR3+Ly6G+ erythroid island macrophages in the mouse. Exp. Hematol. 42, 547–561.e4. 10.1016/j.exphem.2014.03.009 24721610

[B53] Kato G. J. Steinberg M. H. Gladwin M. T. (2017). Intravascular hemolysis and the pathophysiology of sickle cell disease. J. Clin. Invest. 127, 750–760. 10.1172/JCI89741 28248201 PMC5330745

[B54] Kawane K. Fukuyama H. Kondoh G. Takeda J. Ohsawa Y. Uchiyama Y.. (2001). Requirement of DNase II for definitive erythropoiesis in the mouse fetal liver. Science 292, 1546–1549. 10.1126/science.292.5521.1546 11375492

[B55] Kim K. S. Zhang D. L. Kovtunovych G. Ghosh M. C. Ollivierre H. Eckhaus M. A.. (2018). Infused wild-type macrophages reside and self-renew in the liver to rescue the hemolysis and anemia of Hmox1-deficient mice. Blood Adv. 2, 2732–2743. 10.1182/bloodadvances.2018019737 30337301 PMC6199647

[B56] Klei T. R. L. Meinderts S. M. van den Berg T. K. van Bruggen R. (2017). From the cradle to the grave: the role of macrophages in erythropoiesis and erythrophagocytosis. Front. Immunol. 8:73. 10.3389/fimmu.2017.00073 28210260 PMC5288342

[B57] Kohyama M. Ise W. Edelson B. T. Wilker P. R. Hildner K. Mejia C.. (2009). Role for Spi-C in the development of red pulp macrophages and splenic iron homeostasis. Nature 457, 318–321. 10.1038/nature07472 19037245 PMC2756102

[B58] Koury M. J. Bondurant M. C. (1990). Erythropoietin retards DNA breakdown and prevents programmed death in erythroid progenitor cells. Science 248, 378–381. 10.1126/science.2326648 2326648

[B59] Kovtunovych G. Eckhaus M. A. Ghosh M. C. Ollivierre-Wilson H. Rouault T. A. (2010). Dysfunction of the heme recycling system in heme oxygenase 1-deficient mice: Effects on macrophage viability and tissue iron distribution. Blood 116, 6054–6062. 10.1182/blood-2010-03-272138 20844238 PMC3031391

[B60] Kristiansen M. Graversen J. H. Jacobsen C. Sonne O. Hoffman H. J. Law S. K. A.. (2001). Identification of the haemoglobin scavenger receptor. Nature 409, 198–201. 10.1038/35051594 11196644

[B61] Kusakabe M. Hasegawa K. Hamada M. Nakamura M. Ohsumi T. Suzuki H.. (2011). c-Maf plays a crucial role for the definitive erythropoiesis that accompanies erythroblastic island formation in the fetal liver. Blood 118, 1374–1385. 10.1182/blood-2010-08-300400 21628412

[B62] Lee G. Lo A. Short S. A. Mankelow T. J. Spring F. Parsons S. F.. (2006). Targeted gene deletion demonstrates that the cell adhesion molecule ICAM-4 is critical for erythroblastic island formation. Blood 108, 2064–2071. 10.1182/blood-2006-03-006759 16690966 PMC1895542

[B63] Lee G. Spring F. A. Parsons S. F. Mankelow T. J. Peters L. L. Koury M. J.. (2003). Novel secreted isoform of adhesion molecule ICAM-4: potential regulator of membrane-associated ICAM-4 interactions. Blood 101, 1790–1797. 10.1182/blood-2002-08-2529 12406883

[B64] Lee J. C. M. Gimm J. A. Lo A. J. Koury M. J. Krauss S. W. Mohandas N.. (2004). Mechanism of protein sorting during erythroblast enucleation: role of cytoskeletal connectivity. Blood 103, 1912–1919. 10.1182/blood-2003-03-0928 14563645

[B65] Lee S. H. Crocker P. R. Westaby S. Key N. Mason D. Y. Gordon S.. (1988). Isolation and immunocytochemical characterization of human bone marrow stromal macrophages in hemopoietic clusters. J. Exp. Med. 168, 1193–1198. 10.1084/jem.168.3.1193 3049905 PMC2189021

[B66] Leimberg M. J. Prus E. Konijn A. M. Fibach E. (2008). Macrophages function as a ferritin iron source for cultured human erythroid precursors. J. Cell. Biochem. 103, 1211–1218. 10.1002/jcb.21499 17902167

[B67] Lenox L. E. Perry J. M. Paulson R. F. (2005). BMP4 and Madh5 regulate the erythroid response to acute anemia. Blood 105, 2741–2748. 10.1182/blood-2004-02-0703 15591122

[B68] Levin N. Bischoff E. D. Daige C. L. Thomas D. Vu C. T. Heyman R. A.. (2005). Macrophage liver X receptor is required for antiatherogenic activity of LXR agonists. Arterioscler. Thromb. Vasc. Biol. 25, 135–142. 10.1161/01.ATV.0000150044.84012.68 15539622

[B69] Li J. Hale J. Bhagia P. Xue F. Chen L. Jaffray J.. (2014). Isolation and transcriptome analyses of human erythroid progenitors: BFU-E and CFU-E. Blood 124, 3636–3645. 10.1182/blood-2014-07-588806 25339359 PMC4256913

[B70] Li W. Wang Y. Zhao H. Zhang H. Xu Y. Wang S.. (2019). Identification and transcriptome analysis of erythroblastic island macrophages. Blood 134, 480–491. 10.1182/blood.2019000430 31101625 PMC6676133

[B71] Liao C. Hardison R. C. Kennett M. J. Carlson B. A. Paulson R. F. Prabhu K. S. (2018a). Selenoproteins regulate stress erythroid progenitors and spleen microenvironment during stress erythropoiesis. Blood 131, 2568–2580. 10.1182/blood-2017-08-800607 29615406 PMC5992864

[B72] Liao C. Sandeep Prabhu K. Paulson R. F. (2018b). Monocyte-derived macrophages expand the murine stress erythropoietic niche during the recovery from anemia. Blood 132, 2580–2593. 10.1182/blood-2018-06-856831 30322871 PMC6293871

[B73] Lim D. Kim K. S. Jeong J. H. Marques O. Kim H. J. Song M.. (2018). The hepcidin-ferroportin axis controls the iron content of Salmonella-containing vacuoles in macrophages. Nat. Commun. 9:091. 10.1038/s41467-018-04446-8 29844422 PMC5974375

[B74] Lipton J. M. Ellis S. R. (2009). Diamond-Blackfan Anemia: Diagnosis, Treatment, and Molecular Pathogenesis. Hematol. Oncol. Clin. North Am. 23, 261–282. 10.1016/j.hoc.2009.01.004 19327583 PMC2886591

[B75] Lombardi E. Matte A. Risitano A. M. Ricklin D. Lambris J. D. De Zanet D.. (2019). Factor H interferes with the adhesion of sickle red cells to vascular endothelium: a novel disease-modulating molecule. Haematologica 104, 919–928. 10.3324/haematol.2018.198622 30630982 PMC6518911

[B76] Lopez-Yrigoyen M. Yang C. T. Fidanza A. Cassetta L. Taylor A. H. McCahill A.. (2019). Genetic programming of macrophages generates an in vitro model for the human erythroid island niche. Nat. Commun. 10:881. 10.1038/s41467-019-08705-0 30787325 PMC6382809

[B77] Luo B. Gan W. Liu Z. Shen Z. Wang J. Shi R.. (2016). Erythropoeitin signaling in macrophages promotes dying cell clearance and immune tolerance. Immunity 44, 287–302. 10.1016/j.immuni.2016.01.002 26872696

[B78] Mankelow T. J. Spring F. A. Parsons S. F. Brady R. L. Mohandas N. Chasis J. A.. (2004). Identification of critical amino-acid residues on the erythroid intercellular adhesion molecule-4 (ICAM-4) mediating adhesion to α v integrins. Blood 103, 1503–1508. 10.1182/blood-2003-08-2792 14551135

[B79] Miyagawa S. I. Kobayashi M. Konishi N. Sato T. Ueda K. (2000). Insulin and insulin-like growth factor I support the proliferation of erythroid progenitor cells in bone marrow through the sharing of receptors. Br. J. Haematol. 109, 555–562. 10.1046/j.1365-2141.2000.02047.x 10886204

[B80] Miyanishi M. Tada K. Koike M. Uchiyama Y. Kitamura T. Nagata S. (2007). Identification of Tim4 as a phosphatidylserine receptor. Nature 450, 435–439. 10.1038/nature06307 17960135

[B81] Mohandas N. Prenant M. (1978). Three-dimensional model of bone marrow. Blood 51, 633–643. 10.1182/blood.v51.4.633.bloodjournal514633 630113

[B82] Morris L. Crocker P. R. Fraser I. Hill M. Gordon S. (1991). Expression of a divalent cation-dependent erythroblast adhesion receptor by stromal macrophages from murine bone marrow. J. Cell Sci. 99, 141–147. 1757498 10.1242/jcs.99.1.141

[B83] Muckenthaler M. U. Rivella S. Hentze M. W. Galy B. (2017). A red carpet for iron metabolism. Cell 168, 344–361. 10.1016/j.cell.2016.12.034 28129536 PMC5706455

[B84] Nemeth E. Ganz T. (2009). The role of hepcidin in iron metabolism. Acta Haematol. 122, 78–86. 10.1159/000243791 19907144 PMC2855274

[B85] Nemeth E. Tuttle M. S. Powelson J. Vaughn M. D. Donovan A. Ward D. M. V.. (2004). Hepcidin regulates cellular iron efflux by binding to ferroportin and inducing its internalization. Science 306, 2090–2093. 10.1126/science.1104742 15514116

[B86] Nilsson A. Isgaard J. Lindahl A. Dahlström A. Skottner A. Isaksson O. G. P. (1986). Regulation by growth hormone of number of chondrocytes containing IGF-I in rat growth plate. Science 233, 571–574. 10.1126/science.3523759 3523759

[B87] Nimer S. D. (2008). Myelodysplastic syndromes. Blood 111, 4841–4851. 10.1182/blood-2007-08-078139 18467609

[B88] Papavassiliou K. A. Papavassiliou A. G. (2016). Transcription factor drug targets. J. Cell. Biochem. 117, 2693–2696. 10.1002/jcb.25605 27191703

[B89] Paulson R. F. (2019). Epo receptor marks the spot. Blood 134, 413–414. 10.1182/blood.2019001581 31371394 PMC6676130

[B90] Paulson R. F. Ruan B. Hao S. Chen Y. (2020). Stress erythropoiesis is a key inflammatory response. Cells 9:634. 10.3390/cells9030634 32155728 PMC7140438

[B91] Pitchford S. C. Lodie T. Rankin S. M. (2012). VEGFR1 stimulates a CXCR4-dependent translocation of megakaryocytes to the vascular niche, enhancing platelet production in mice. Blood 120, 2787–2795. 10.1182/blood-2011-09-378174 22653973

[B92] Porcu S. Manchinu M. F. Marongiu M. F. Sogos V. Poddie D. Asunis I.. (2011). Klf1 affects DNase II-Alpha expression in the central macrophage of a fetal liver erythroblastic island: a non-cell-autonomous role in definitive erythropoiesis. Mol. Cell. Biol. 31, 4144–4154. 10.1128/mcb.05532-11 21807894 PMC3187365

[B93] Poss K. D. Tonegawa S. (1997). Heme oxygenase 1 is required for mammalian iron reutilization. Proc. Natl. Acad. Sci. U.S.A. 94, 10919–10924. 10.1073/pnas.94.20.10919 9380735 PMC23531

[B94] Qu X. Zhang S. Wang S. Wang Y. Li W. Huang Y.. (2018). TET2 deficiency leads to stem cell factor–dependent clonal expansion of dysfunctional erythroid progenitors. Blood 132, 2406–2417. 10.1182/blood-2018-05-853291 30254129 PMC6265651

[B95] Rajbhandari N. Lin W. chi Wehde B. L. Triplett A. A. Wagner K. U. (2017). Autocrine IGF1 signaling mediates pancreatic tumor cell dormancy in the absence of oncogenic drivers. Cell Rep. 18, 2243–2255. 10.1016/j.celrep.2017.02.013 28249168 PMC5369772

[B96] Ramos P. Casu C. Gardenghi S. Breda L. Crielaard B. J. Guy E.. (2013). Macrophages support pathological erythropoiesis in polycythemia vera and β-thalassemia. Nat. Med. 19, 437–445. 10.1038/nm.3126 23502961 PMC3618568

[B97] Rantakari P. Jäppinen N. Lokka E. Mokkala E. Gerke H. Peuhu E.. (2016). Fetal liver endothelium regulates the seeding of tissue-resident macrophages. Nature 538, 392–396. 10.1038/nature19814 27732581

[B98] Rhodes M. M. Kopsombut P. Bondurant M. C. Price J. O. Koury M. J. (2008). Adherence to macrophages in erythroblastic islands enhances erythroblast proliferation and increases erythrocyte production by a different mechanism than erythropoietin. Blood 111, 1700–1708. 10.1182/blood-2007-06-098178 17993612 PMC2214751

[B99] Sadahira Y. Yoshino T. Monobe Y. (1995). Very late activation antigen 4-vascular cell adhesion molecule 1 interaction is involved in the formation of erythroblastic Islands. J. Exp. Med. 181, 411–415. 10.1084/jem.181.1.411 7528776 PMC2191848

[B100] Sathyanarayana P. Dev A. Fang J. Houde E. Bogacheva O. Bogachev O.. (2008). EPO receptor circuits for primary erythroblast survival. Blood 111, 5390–5399. 10.1182/blood-2007-10-119743 18349318 PMC2396729

[B101] Sawada K. Krantz S. B. Dessypris E. N. Koury S. T. Sawyer S. T. (1989). Human colony-forming units-erythroid do not require accessory cells, but do require direct interaction with insulin-like growth factor I and/or insulin for erythroid development. J. Clin. Invest. 83, 1701–1709. 10.1172/JCI114070 2651478 PMC303879

[B102] Schwarz K. Iolascon A. Verissimo F. Trede N. S. Horsley W. Chen W.. (2009). Mutations affecting the secretory COPII coat component SEC23B cause congenital dyserythropoietic anemia type II. Nat. Genet. 41, 936–940. 10.1038/ng.405 19561605

[B103] Seu K. G. Papoin J. Fessler R. Hom J. Huang G. Mohandas N.. (2017). Unraveling macrophage heterogeneity in erythroblastic islands. Front. Immunol. 8:1140. 10.3389/fimmu.2017.01140 28979259 PMC5611421

[B104] Soni S. Bala S. Gwynn B. Sahr K. E. Peters L. L. Hanspal M. (2006). Absence of erythroblast macrophage protein (Emp) leads to failure of erythroblast nuclear extrusion. J. Biol. Chem. 281, 20181–20189. 10.1074/jbc.M603226200 16707498

[B105] Tay J. Bisht K. McGirr C. Millard S. M. Pettit A. R. Winkler I. G.. (2020). Imaging flow cytometry reveals that granulocyte colony-stimulating factor treatment causes loss of erythroblastic islands in the mouse bone marrow. Exp. Hematol. 82, 33–42. 10.1016/j.exphem.2020.02.003 32045657

[B106] Toda S. Nishi C. Yanagihashi Y. Segawa K. Nagata S. (2015). Clearance of apoptotic cells and pyrenocytes, in Current Topics in Developmental Biology, ed Steller H. (New York, NY: Academic Press Inc.) 267–295. 10.1016/bs.ctdb.2015.07.017 26431571

[B107] Trenor C. C. Campagna D. R. Sellers V. M. Andrews N. C. Fleming M. D. (2000). The molecular defect in hypotransferrinemic mice. Blood 96, 1113–1118. 10.1182/blood.v96.3.1113.015k03_1113_1118 10910930

[B108] Victor A. R. Nalin A. P. Dong W. McClory S. Wei M. Mao C.. (2017). IL-18 drives ILC3 proliferation and promotes IL-22 production via NF-κB. J. Immunol. 199, 2333–2342. 10.4049/jimmunol.1601554 28842466 PMC5624342

[B109] Vinchi F. (2018). Targeting bone marrow niche macrophages. HemaSphere 2:e148. 10.1097/hs9.0000000000000148 30887011 PMC6407793

[B110] Vinchi F. Da Silva M. C. Ingoglia G. Petrillo S. Brinkman N. Zuercher A.. (2016). Hemopexin therapy reverts heme-induced proinflammatory phenotypic switching of macrophages in a mouse model of sickle cell disease. Blood 127, 473–486. 10.1182/blood-2015-08-663245 26675351 PMC4850229

[B111] Wang J. Hayashi Y. Yokota A. Xu Z. Zhang Y. Huang R.. (2018). Expansion of EPOR-negative macrophages besides erythroblasts by elevated EPOR signaling in erythrocytosis mouse models. Haematologica 103, 69–79. 10.3324/haematol.2017.172775 29051279 PMC5777189

[B112] Wei Q. Boulais P. E. Zhang D. Pinho S. Tanaka M. Frenette P. S. (2019). Maea expressed by macrophages, but not erythroblasts, maintains postnatal murine bone marrow erythroblastic islands. Blood 133, 1222–1232. 10.1182/blood-2018-11-888180 30674470 PMC6418477

[B113] Weiskopf K. (2017). Cancer immunotherapy targeting the CD47/SIRPα axis. Eur. J. Cancer 76, 100–109. 10.1016/j.ejca.2017.02.013 28286286

[B114] Wickramasinghe S. N. Wood W. G. (2005). Advances in the understanding of the congenital dyserythropoietic anaemias. Br. J. Haematol. 131, 431–446. 10.1111/j.1365-2141.2005.05757.x 16281933

[B115] Wojchowski D. M. Menon M. P. Sathyanarayana P. Fang J. Karur V. Houde E.. (2006). Erythropoietin-dependent erythropoiesis: new insights and questions. Blood Cells Mol. Dis. 36, 232–238. 10.1016/j.bcmd.2006.01.007 16524748

[B116] Wu X. Dao Thi V. L. Huang Y. Billerbeck E. Saha D. Hoffmann H. H.. (2018). Intrinsic immunity shapes viral resistance of stem cells. Cell 172, 423–438.e25. 10.1016/j.cell.2017.11.018 29249360 PMC5786493

[B117] Xue L. Galdass M. Gnanapragasam M. N. Manwani D. Bieker J. J. (2014). Extrinsic and intrinsic control by EKLF (KLF1) within a specialized erythroid niche. Dev 141, 2245–2254. 10.1242/dev.103960 24866116 PMC4034424

[B118] Yan H. Hale J. Jaffray J. Li J. Wang Y. Huang Y.. (2018). Developmental differences between neonatal and adult human erythropoiesis. Am. J. Hematol. 93, 494–503. 10.1002/ajh.25015 29274096 PMC5842122

[B119] Yan H. Wang Y. Qu X. Li J. Hale J. Huang Y.. (2017). Distinct roles for TET family proteins in regulating human erythropoiesis. Blood 129, 2002–2012. 10.1182/blood-2016-08-736587 28167661 PMC5383871

[B120] Yeo J. H. McAllan B. M. Fraser S. T. (2016). Scanning electron microscopy reveals two distinct classes of erythroblastic Island isolated from adult mammalian bone marrow. Microsc. Microanal. 22, 368–378. 10.1017/S1431927616000155 26898901

[B121] Yokoyama T. Etoh T. Kitagawa H. Tsukahara S. Kannan Y. (2003). Migration of erythroblastic islands toward the sinusoid as erythroid maturation proceeds in rat bone marrow. J. Vet. Med. Sci. 65, 449–452. 10.1292/jvms.65.449 12736425

[B122] Yokoyama T. Kitagawa H. Takeuchi T. Tsukahara S. Kannan Y. (2002). No apoptotic cell death of erythroid cells of erythroblastic islands in bone marrow of healthy rats. J. Vet. Med. Sci. 64, 913–919. 10.1292/jvms.64.913 12419868

[B123] Yoshida H. Kawane K. Koike M. Mori Y. Uchiyama Y. Nagata S. (2005). Phosphatidylserine-dependent engulfment by macrophages of nuclei from erythroid precursor cells. Nature 437, 754–758. 10.1038/nature03964 16193055

[B124] Yu Y. Jiang L. Wang H. Shen Z. Cheng Q. Zhang P.. (2020). Hepatic transferrin plays a role in systemic iron homeostasis and liver ferroptosis. Blood 136, 726–739. 10.1182/BLOOD.2019002907 32374849 PMC7414596

[B125] Zhang D. L. Ghosh M. C. Ollivierre H. Li Y. Rouault T. A. (2018). Ferroportin deficiency in erythroid cells causes serum iron deficiency and promotes hemolysis due to oxidative stress. Blood 132, 2078–2087. 10.1182/blood-2018-04-842997 30213870 PMC6236465

[B126] Zhang Z. Zhang F. An P. Guo X. Shen Y. Tao Y.. (2011). Ferroportin1 deficiency in mouse macrophages impairs iron homeostasis and inflammatory responses. Blood 118, 1912–1922. 10.1182/blood-2011-01-330324 21705499

[B127] Zhang Z. Zhang F. Guo X. An P. Tao Y. Wang F. (2012). Ferroportin1 in hepatocytes and macrophages is required for the efficient mobilization of body iron stores in mice. Hepatology 56, 961–971. 10.1002/hep.25746 22473803

