# Corrigendum: Erythroblastic Island Macrophages Shape Normal Erythropoiesis and Drive Associated Disorders in Erythroid Hematopoietic Diseases

**DOI:** 10.3389/fcell.2022.857022

**Published:** 2022-05-13

**Authors:** Wei Li, Rongqun Guo, Yongping Song, Zhongxing Jiang

**Affiliations:** Department of Hematology, The First Affiliated Hospital of Zhengzhou University, Zhengzhou, China

**Keywords:** EBI macrophages, erythropoiesis, central macrophages, EPOR, erythroid hematopoietic disorders, β-thalassemia, polycythemia vera

An author name was incorrectly spelled as “Zhongxin Jiang”. The correct spelling is “Zhongxing Jiang”.

There is an error in the Funding statement. Please remove “Natural Science Foundation of China (82000112)”.

The authors apologize for this error and state that this does not change the scientific conclusions of the article in any way. The original article has been updated.

